# Genomic and Structural Investigation of Mutations in Biotinidase (BTD) Gene Deficiency in Greater Middle Eastern Cohort: Insights from Molecular Dynamics Study

**DOI:** 10.3390/biomedicines13092210

**Published:** 2025-09-09

**Authors:** Faisal E. Ibrahim, BalaSubramani Gattu Linga, Muthanna Samara, Jameela Roshanuddin, Salma Younes, Gheyath K. Nasrallah, Hatem Zayed, M. Walid Qoronfleh, Sawsan G. A. A. Mohammed, Dalia El Khoury, Dinesh Velayutham, Ghassan Abdoh, Hilal Al Rifai, Nader Al-Dewik

**Affiliations:** 1 Department of Research, Women’s Wellness and Research Center, Hamad Medical Corporation (HMC), P.O. Box 3050, Doha 0974, Qatar; fibrahim23@hamad.qa (F.E.I.); blinga@hamad.qa (B.G.L.); juddin1@hamad.qa (J.R.); 2 Translational and Precision Medicine Research, Women’s Wellness and Research Center (WWRC), Hamad Medical Corporation (HMC), Doha 0974, Qatar; 3 Translational Research Institute (TRI), Hamad Medical Corporation (HMC), Doha 3050, Qatar; 4 Department of Psychology, Kingston University London, Penrhyn Road, London KT1 2EE, UK; m.samara@kingston.ac.uk; 5 Qatar Preterm and Precision Medicine Research Clinic, Women’s Wellness and Research Center, Hamad Medical Corporation (HMC), P.O. Box 3050, Doha 0974, Qatar; 6 Department of Biomedical Science, College of Health Sciences, Member of QU Health, Qatar University, Doha 2713, Qatar; sy1203986@student.qu.edu.qa (S.Y.); gheyath.nasrallah@qu.edu.qa (G.K.N.); hatem.zayed@qu.edu.qa (H.Z.); 7 Biomedical Research Center, QU Health, Qatar University, Doha 2713, Qatar; 8 Healthcare Research & Policy Division, Q3 Research Institute (QRI), Ann Arbor, MI 48197, USA; 9 QU Health, College of Medicine, Qatar University, Doha 2713, Qatar; sawsan@qu.edu.qa; 10 Department of Family Relations and Applied Nutrition, University of Guelph, 50 Stone Road East, Guelph, ON N1G2W1, Canada; delkhour@uoguelph.ca; 11 Liberal Arts and Science (LAS), Hamad Bin Khalifa University (HBKU), P.O. Box 34110, Doha 34110, Qatar; dinesh.peace@gmail.com; 12 Department of Neonatology, Neonatal Intensive Care Unit (NICU), Newborn Screening Unit, Women’s Wellness and Research Center (WWRC), Hamad Medical Corporation (HMC), Doha 3050, Qatar; gabdoh@hamad.qa; 13 Genomics and Precision Medicine (GPM), College of Health & Life Science (CHLS), Hamad Bin Khalifa University (HBKU), Doha 34110, Qatar; halrifai@hamad.qa; 14 Faculty of Health and Social Care Sciences, Kingston University, St. George’s University of London, London KT1 2EE, UK

**Keywords:** biotinidase deficiency, genotype–phenotype correlation, Qatar Genome Program, single-nucleotide polymorphism, protein stability, molecular dynamics

## Abstract

**Background**: Biotinidase deficiency (BD) is a common autosomal recessive metabolic disorder in Qatar and the Arab world. It is treatable if detected early, making it essential to understand the genetic variants involved. This study aimed to investigate the carrier frequency of BD-related variants in a healthy Qatari population, reflecting the genetic landscape of the broader Middle Eastern region; classify them using bioinformatics tools; and compare findings with global datasets. **Methods**: Whole-genome sequencing data from 14,669 participants in the Qatar Genome Program (QGP), a multiethnic cohort including Qatari nationals and long-term residents (≥15 years), were analyzed to identify BTD variants. A total of 723, including 653 single-nucleotide polymorphisms (SNPs) and 70 structural variants (SVs) in BTD associated with BD, were screened against the Qatari cohort and compared with international data. *In silico* tools were used to assess variant pathogenicity, conservation, and protein stability. Molecular dynamics (MD) simulations were performed to evaluate structural and functional changes in the BTD. **Results**: A total of 80 SNPs and 3 SVs were identified, among which 21 variants (19 SNPs and 2 SVs) were classified as pathogenic or likely pathogenic, according to ClinVar. The carrier frequency of BTD-related variants in Qatar was 1:20, primarily driven by rs13078881 (D444H). Molecular dynamics (MD) simulations revealed significant conformational changes with H323R, D444H, and P497S, which demonstrated increased flexibility (higher RMSD/RMSF and PCA trace values). Additionally, R209C and D444H showed reduced compactness (higher Rg) and distinct energy minima, suggesting altered conformational states. **Conclusions**: This study demonstrates a high carrier frequency of pathogenic BTD variants in the Qatari population, underscoring the need to integrate these SNPs and SVs into the national genomic neonatal screening program (gNBS) for enhanced early detection and treatment strategies. The mild structural deviations observed in the D444H mutant through MD simulations may explain its association with milder clinical phenotypes of BTD, offering valuable insights for personalized therapeutic approaches.

## 1. Introduction

Biotin is a crucial coenzyme for several carboxylases, aiding in essential metabolic processes by forming biotinylated monocarboxylates [1]. Biotinidase (BTD) (EC 3.5.1.12) hydrolyzes biocytin, releasing biotin for recycling. Biotinidase deficiency (BD) is an autosomal recessive disorder caused by mutations in the BTD gene, resulting in reduced or absent BTD activity, impairing biotin recycling [2]. The BTD gene contains four exons [3], and its protein sequence shares homology with bacterial amidases and nitrilases, with Cys245 and the YRK210-212 sequence being crucial for enzyme function [4,5]. BTD symptoms range from mild (hypotonia, skin rashes, and hair loss) to severe, resulting in seizures, developmental delays, and sensory loss without treatment [6,7,8,9,10,11]. Early diagnosis through neonatal screening and biotin therapy can prevent or alleviate most symptoms [8,9,12]. In severe cases, biotin therapy (5–20 mg daily) can reverse symptoms, although developmental delays and hearing loss may persist if present before treatment [11,13].

The worldwide prevalence of BTD ranges from 1:40,000 to 1:60,000 births [9,14,15] with higher incidence in countries with a high rate of consanguinity, such as Saudi Arabia, the United Arab Emirates, and Qatar [16,17,18,19,20,21,22,23,24,25,26,27,28,29,30,31,32,33,34,35,36,37]. The incidence is estimated at 1:28,316, 1:7116, 1:9000, and 1:20,000 in Saudi Arabia, Türkiye, Brazil, and the UAE, respectively [11,38,39,40]. Consanguinity and founder mutations remain major contributors to the higher frequency of BTD in these regions [41]. Diagnosis is typically made through prenatal screening or biochemical testing [11], while whole-exome sequencing offers precise genetic diagnosis with over 99% sensitivity [42,43]. Newborn screening (NBS) for BTD was introduced in the United States in 1984 and became mandatory in 1986 [44,45]. Over the past 40 years, clinical data have highlighted BTD’s key features [46]. The BTD gene, located on chromosome 3p25, has been reported to carry 165 variants, of which 155 are classified as pathogenic, including missense, nonsense, and deletion mutations [47,48,49,50,51,52,53,54,55,56,57].Non-synonymous single-nucleotide polymorphisms (nsSNPs) in coding regions can affect protein function, influencing disease susceptibility [58,59,60,61]. Advances in computational biology streamline the evaluation of nsSNPs and prediction of their impact on protein function [62,63,64]. Machine learning techniques further enhance these predictions [65,66,67,68,69,70]. However, research on BD-causing nsSNPs in Arab populations remains limited [16,17,25,71,72,73,74,75,76,77,78,79,80]. While some studies identified common pathogenic variants in countries like Saudi Arabia and the UAE [16,17,72,74,75,77,78], there is no comprehensive research on potential pathogenic BTD variants in the Qatari population.

This study assessed the carrier frequency of BTD-related variants in a healthy cohort of 14,669 individuals from the Qatar Genome Program (QGP). The QGP represents a multiethnic population from the Greater Middle East, including Qatari nationals and long-term residents (≥15 years), providing a valuable snapshot of regional genetic diversity. To provide broader context, we compared these findings with global genomic datasets to gain insights into both the regional and worldwide genetic variability of BD. Additionally, we performed a comprehensive computational screening of a deleterious single-nucleotide polymorphism (SNP) and structural variant (SV) dataset to assess their structural impact on the BTD protein.

## 2. Materials and Methods

**Figure 1:**
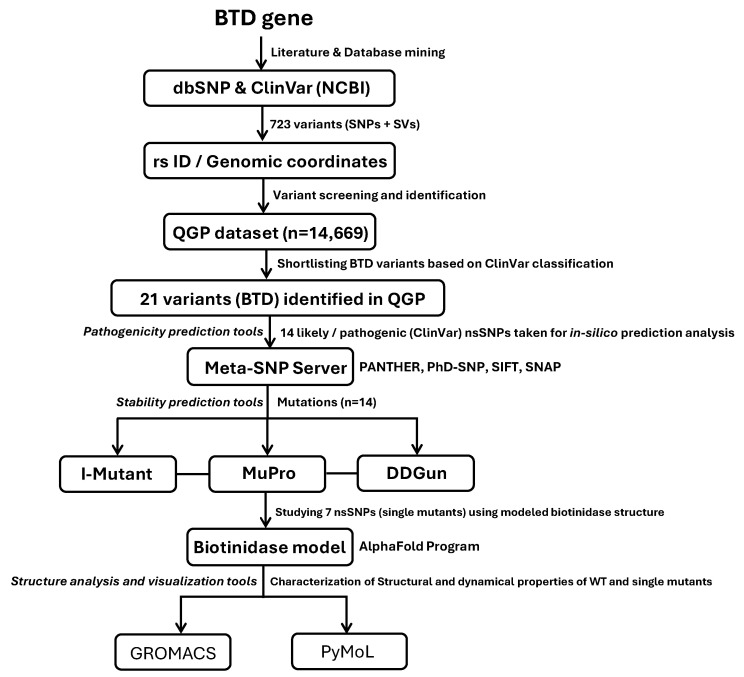
Schematic representation of BTD SNPs and SVs screening against QGP cohort and its computational validation. The workflow outlines the identification and computational analysis of BTD variants. Literature and database mining (dbSNP, ClinVar) identified 723 SNPs and SVs, which were mapped to rsIDs and genomic coordinates. These variants were screened against the Qatar Genome Program (QGP) dataset (n = 14,669), leading to the identification of 83 variants. Based on ClinVar annotations, 21 BTD variants were initially shortlisted, of which 7 likely pathogenic or pathogenic nonsynonymous SNPs (nsSNPs) were subjected to *in silico* pathogenicity prediction using the Meta-SNP server (comprising PANTHER, PhD-SNP, SIFT, and SNAP). All selected variants were predicted to be pathogenic by MutationTaster, except for the intronic variant rs530872564, which was classified as benign. Protein stability analysis was performed using I-Mutant, MuPro, and DDGun. Seven nsSNPs were structurally modeled using the AlphaFold-predicted *BTD* structure, followed by molecular dynamics simulations in GROMACS to assess their structural and dynamic properties. PyMOL was used for visualization and structural analysis, providing insights into mutation-induced conformational changes in *BTD*.

A total of 723 variants (653 SNPs and 70 SVs) associated with the BTD gene, comprising pathogenic, likely pathogenic, and conflicting classifications, were compiled from literature sources and variant databases, including PubMed, Google Scholar, and ClinVar [81] (Table S1). A schematic representation of these methods is depicted in Figure 1. This study includes a healthy population from the Qatar Genome Program (QGP) dataset, which comprises 14,669 participants and represents a multiethnic cohort from greater Middle Eastern countries (e.g., Egypt, Gulf Council Countries, Iran, Türkiye, Palestine, India subcontinent). The study includes the genetic data from individuals who are Qatari nationals and long-term residents in Qatar (≥15 years). Therefore, the population serves as a valuable representation of the larger Middle Eastern population [19].

These SNPs and SVs were screened against a cohort of 14,669 individuals (QGP dataset) to compute allelic, genotypic frequencies and compare with global populations. The genotype frequencies were provided for comparison with an international population (IP) dataset of 2504 individuals [82]. Computational tools like CADD [83] and PhyloP [84] were used to annotate the variants’ effects. Allelic and genotypic frequency data were retrieved from datasets, including ExAC 0.3 [85], 1000 Genomes [86], and esp6500si [85].

We screened 723 known BTD-associated variants against the QGP dataset and identified 83 variants that are present in the cohort, including 21 classified as pathogenic or likely pathogenic (P/LP). MutationTaster was employed to predict the pathogenicity of all shortlisted BTD variants (n = 21). A further 7 high-frequency missense variants were shortlisted from these 21 variants, and our *in silico* structural analysis focused on these 7 high-frequency missense variants amenable to molecular modeling. Frameshift, nonsense, and large SVs were excluded due to current limitations in structure prediction tools. While novel variants may exist in the QGP dataset, their analysis was beyond the current study’s scope (see Figure 1).

### 2.1. Pathogenicity and Evolutionary Conservation Analysis

MutationTaster was employed to predict the pathogenicity of all shortlisted BTD variants (n = 21). This tool integrates information on evolutionary conservation, splice-site alterations, protein feature loss, and the functional impact of sequence changes to classify variants as disease-causing or benign [87]. To complement this, evolutionary conservation analysis was performed using the ConSurf server [88], which evaluates the evolutionary rate of each amino acid in the BTD protein. Conservation scores range from 1 (highly variable) to 9 (highly conserved), enabling the identification of residues with potential structural or functional importance [88].

### 2.2. Variant Prioritization and In Silico Prediction Tools

From the 21 variants, a subset of 14 missense (nsSNP) variants classified as pathogenic or likely pathogenic in ClinVar were prioritized for downstream pathogenicity and stability prediction. Four *in silico* tools, namely Meta-SNP [89,90,91], I-mutant-2.0 [92], MuPro [93], and DDGun [94], were employed to assess the impact of amino acid mutations on the structure and function of proteins. These tools utilize machine learning algorithms and thermodynamic modeling to predict the effects of missense mutations on protein stability and pathogenic potential.

### 2.3. Prediction of Deleterious nsSNPs

To evaluate the pathogenic potential of the 14 prioritized missense variants, the BTD protein sequence (UniProt ID: P43251) was analyzed using the Meta-SNP server. This platform integrates multiple predictive algorithms—PANTHER [95], PhD-SNP [96], SIFT [97], SNAP [98], and Meta-SNP—to generate a consensus prediction. A Meta-SNP score above 0.5 indicates a likely disease-causing mutation. The tool demonstrated a reported overall accuracy of 79% and a Matthews correlation coefficient (MCC) of 0.59, with optimal performance (87% accuracy, 0.73 MCC) when all component predictors concur [89].

### 2.4. Prediction of Destabilizing nsSNPs

The same 14 missense variants were analyzed to predict their effects on protein structural stability using three established tools: Mutant 2.0 [92], MUpro 1.1 [93], and DDGun [94]. These methods calculate the change in Gibbs free energy (ΔΔG) between wild-type (WT) and mutant proteins. Negative ΔΔG values suggest destabilizing mutations, while positive values indicate stabilizing effects. I-Mutant 2.0 accepts either a protein sequence or structure as input and classifies mutations accordingly. Similarly, MuPro and DDGun employ machine-learning-based approaches to assess stability effects based on amino acid substitution patterns.

### 2.5. In Silico Structural Modeling and Molecular Dynamics Simulation

From the 14 missense variants evaluated in Section 2.3 and Section 2.4, a final set of 7 high-priority missense variants—classified as pathogenic/likely pathogenic and exhibiting higher allele frequency in the Qatari cohort—were selected for structural modeling and molecular dynamics (MD) simulations. These variants were modeled to assess their impact on BTD protein structure, conformational flexibility, and overall stability under physiological conditions.

### 2.6. Modelled Structure of Biotinidase

The WT structure of the BTD protein was modeled using AlphaFold, and seven pathogenic missense mutations were subsequently introduced via site-directed mutagenesis using PyMOL [99] and Swiss PDB Viewer [99,100,101]. Following mutagenesis, each mutant structure underwent energy minimization to resolve steric clashes and optimize local conformations prior to downstream validation and simulation. Structural quality assessment was performed using ERRAT for non-bonded interaction analysis [102], PROCHECK for stereochemical quality was validated through Ramachandran plots [103,104], and ProSA-web for evaluating overall model accuracy based on statistical potentials was performed [105]. Additionally, Biopython was used to extract backbone dihedral angles (φ, ψ), and matplotlib with seaborn was employed to visualize Ramachandran contour density plots and residue-specific conformational preferences across structural classes.

### 2.7. Secondary Structure and Physicochemical Properties

Secondary structure elements and physicochemical parameters of the BTD-WT and mutant proteins were analyzed using the PDBsum server and ProtParam tool [106,107]. Key parameters such as isoelectric point (pI), aliphatic index, GRAVY (hydropathicity), and instability index were calculated to gain insights into the structural and solubility characteristics of the proteins.

### 2.8. Salt Bridge Analysis

To assess the role of electrostatic interactions in protein stability, salt bridge interactions between oppositely charged residues were analyzed using the ESBRI web server [108,109]. Salt bridge interactions were re-evaluated for both WT and mutant proteins using the ESBRI server following energy minimization to assess mutation-induced changes in electrostatic stability.

### 2.9. Molecular Dynamics (MD) Simulation Studies

MD simulations were carried out for both WT and mutant BTD structures using GROMACS 2024.2 [108]. Systems were solvated in a cubic box with explicit water molecules, neutralized with Na^+^ counterions, and subjected to energy minimization. Equilibration was conducted for 100 ps, followed by 50 ns production runs. Temperature and pressure were controlled using the Berendsen thermostat and LINCS algorithm for bond constraints [110,111]. Long-range electrostatics were calculated using the particle mesh Ewald (PME) method [112]. Simulation trajectories were analyzed for structural stability and flexibility using metrics such as RMSD, RMSF, radius of gyration (Rg), solvent-accessible surface area (SASA), and hydrogen bond count [113].

## 3. Results

### 3.1. Analysis of BTD Variants in Qatar Population

A total of 83 variants in the *BTD* gene were identified through whole-genome sequencing of 14,669 participants in the Qatar Genome Program (QGP) (Table S2). Among these, 21 variants were classified as pathogenic or likely pathogenic (P/LP) based on ClinVar annotations. This subset included 15 missense single-nucleotide variants (nsSNPs), 2 nonsense variants (Y57Ter and Q156Ter), 2 frameshift variants (G34fs and R164fs), 1 intronic variant (rs530872564), and 1 synonymous variant. To assess allele distribution, genotype frequencies of these 21 P/LP variants were compared with those from an international reference cohort (n = 2504; 1000 Genomes Project). The QGP dataset demonstrated a significantly elevated carrier frequency of 1:20, with 25 individuals homozygous for pathogenic variants (22 in D444H, 2 in H323R, and 1 in R209C) and 756 heterozygous carriers across the 21 loci (Table 1). The allelic frequency for both H323R and A478T was found to be significantly higher in IP (Table S3). The differences in D444H variant frequencies between the QGP and IP cohorts, for variant homozygosity vs. heterozygosity and wild-type, were not statistically significant. In contrast, only 121 heterozygous cases were observed in the international dataset, underscoring a higher burden of BTD-associated pathogenic variants in the Qatari population. Among the 15 missense variants, three (I108V, G480E, and L535V) showed conflicting ClinVar classifications but were retained for preliminary analysis due to their protein-altering potential. Conversely, the intronic variant rs530872564 was excluded from further functional analysis due to its non-coding nature and benign prediction.

Variants predicted to cause loss of function, including the two frameshift variants (G34fs, R164fs), nonsense mutations (Y57Ter, Q156Ter), and structural variants (rs765906887 and rs397514365), were not subjected to *in silico* structural modeling, as tools used in subsequent analyses (e.g., Meta-SNP, I-Mutant 2.0) were not optimized for truncating variants. Nonetheless, these mutations retain clinical significance based on established associations with enzyme deficiency. Finally, six additional missense variants (A478T, T479M, T532M, D543G, I108V, and L535V) were excluded from downstream modeling due to extremely low allele frequency (<0.15%) in both Qatari and international populations and/or limited evidence of pathogenicity. These exclusions ensured that downstream modeling focused on variants with greater clinical relevance and population impact.

### 3.2. Conservation Analysis

**Figure 2:**
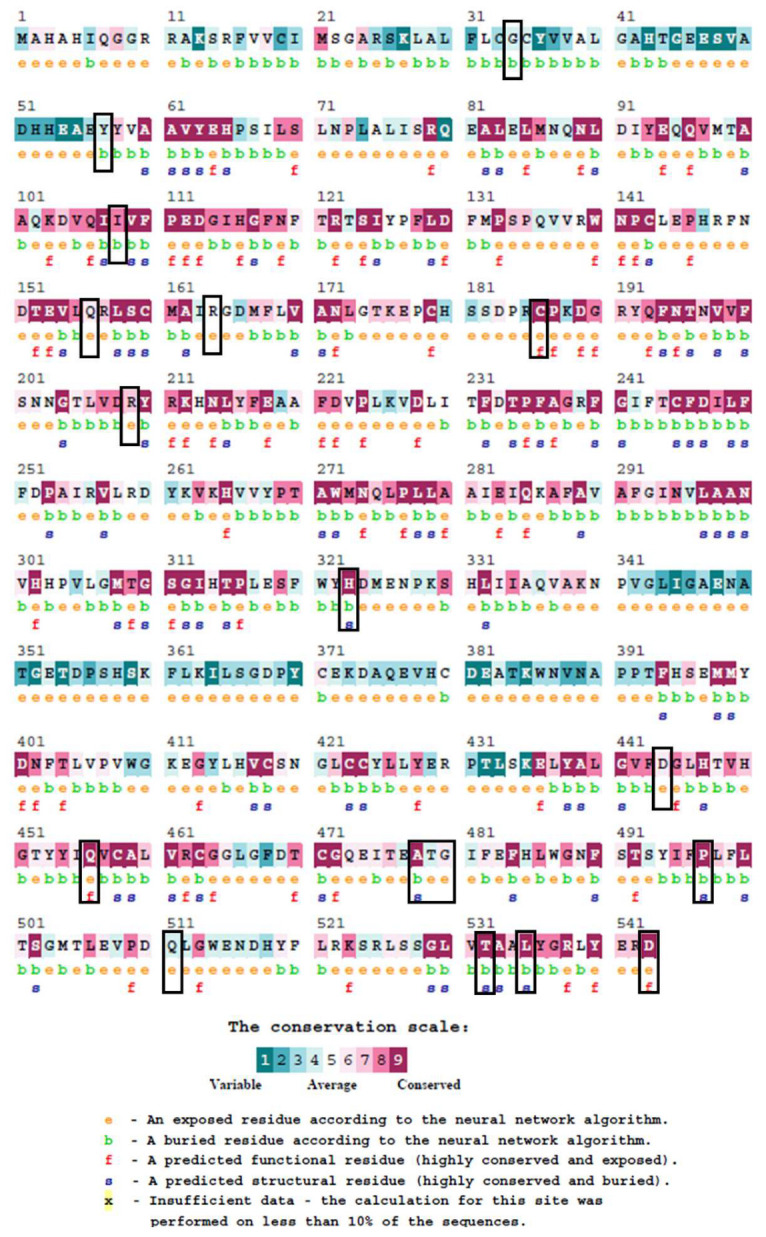
Conservation analysis of BTD protein sequence using ConSurf. The output, obtained from the web server, displays sequence conservation for ClinVar-reported pathogenic and likely pathogenic mutants. Conservation is represented by color, with highly conserved positions marked in purple (score ≥8) and moderately conserved residues in blue to light purple (scores 5–7). Residues C186, R209, H323, Q456, and P497 are highly conserved, located in exposed regions, and are likely to play critical functional roles. Additionally, D444 and Q511 have moderate conservation scores (>5) and are also present in exposed regions. Other analyzed mutant positions (e.g., A478T, T479M, D543G, and L535V) demonstrate varying conservation and structural impact, contributing to the pathogenic potential of these mutations.

The ConSurf analysis of the BTD protein provided detailed insights into the evolutionary conservation, structural roles, and potential functional importance of all analyzed residues. Highly conserved residues, including C186, H323, Q456, P497, A478, T532, L535, and D543, were assigned the maximum conservation score of 9. Importantly, all residues prioritized for downstream structural modeling (C186, R209, H323, D444, Q456, P497, and Q511) exhibited both high conservation (scores 6–9) and reliable confidence intervals (CI ≤ 1), ensuring the robustness of their conservation assignments (Figure 2). Residues with lower confidence intervals or extremely low genotype frequencies (≤0.01%)—such as A478, T532, L535, and D543—were de-emphasized or excluded from downstream analyses to maintain population relevance and structural interpretability. C186, Q456, and D444 were predicted to be exposed and functional residues, suggesting potential roles in substrate recognition or enzymatic activity, while H323, P497, and Q511 were predicted to be buried and structural, consistent with their importance in maintaining protein stability. R209, though moderately conserved (score 7), is situated adjacent to the YRK210–212 motif near the catalytic K212, warranting its inclusion in downstream analysis due to potential functional implications. Conversely, residues such as A478, T532, and L535, despite their high conservation scores, were deprioritized due to their very low allele frequency and limited clinical relevance in the studied populations. Moderately conserved residues (I108, Q156, R164, T479, and G480; scores 6–8) were not prioritized, as mutations at these sites are generally less likely to cause substantial functional disruption compared to the high-confidence variants. Lower conservation residues (G34, Y57) were also noted but given minimal weight unless located in key structural regions. Overall, these analyses highlight that mutations at C186, R209, H323, D444, Q456, P497, and Q511, supported by high conservation confidence, represent the most functionally significant variants, while other conserved residues (e.g., A478, T532) were given less weight due to population frequency and limited structural impact.

### 3.3. Pathogenicity and Stability Prediction

To assess the pathogenic potential of BTD missense variants, Meta-SNP analysis was performed on 14 prioritized nsSNPs (Table 2).

Variants with a Meta-SNP score > 0.7 were considered highly likely to be disease-causing. The highest scoring variant was Q456H (rs80338685) with a score of 0.825, indicating strong predicted pathogenicity. This was closely followed by R209C (0.819), D444H (0.811), C186Y (0.799), and T532M (0.720), all of which demonstrated robust associations with BD. Although P497S (0.697) fell slightly below the high pathogenicity threshold, it showed consistent deleterious predictions across all four integrated tools—PANTHER, PhD-SNP, SIFT, and SNAP—suggesting a likely impact on protein function. As such, it remains a strong candidate for further investigation despite its marginal score. In contrast, A478T (0.535) exhibited inconsistent predictions across tools. While PANTHER and SIFT suggested potential pathogenicity, PhD-SNP and SNAP classified it as neutral. This variant may act as a modifier allele, potentially influencing phenotype in the presence of other pathogenic mutations or environmental factors. Variants such as Q511E (0.156), T479M (0.212), G480E (0.260), H323R (0.315), D543G (0.356), L535V (0.361), and I108V (0.425) showed low Meta-SNP scores, indicating a lower probability of being disease-causing. These results are in line with their ClinVar classifications as benign, likely benign, or of uncertain significance, emphasizing the need for cautious interpretation and possible functional validation. Overall, the top-ranking variants—Q456H > R209C > D444H > C186Y > T532M—exhibited high reliability indices (RI), indicating consistent and confident predictions. These mutations were prioritized for downstream structural modeling and dynamic simulation due to their combined pathogenicity potential and population relevance.

To investigate whether these missense mutations affect protein stability, we employed I-Mutant 2.0, MuPro, and DDGun to predict changes in Gibbs free energy (ΔΔG) (Table 3). Negative ΔΔG values indicate destabilizing effects, while positive values suggest stabilizing impacts on protein structure. Eight variants—I108V, C186Y, R209C, D444H, Q456H, P497S, L535V, and D543G—were consistently predicted to be destabilizing across all three tools, reinforcing their potential pathogenicity. L535V (ΔΔG ≈ −2.91 kcal/mol) and P497S (ΔΔG ≈ −1.65 kcal/mol) demonstrated the most pronounced destabilizing effects, supporting the hypothesis that these substitutions may impair protein folding or enzymatic function. Conversely, H323R, G480E, and Q511E were predicted to be stabilizing, with positive ΔΔG values (+0.42, +0.43, and +0.26 kcal/mol, respectively). A478T, T479M, and T532M yielded neutral or near-zero ΔΔG values, suggesting minimal influence on protein structural integrity. This integrative computational approach—combining pathogenicity and stability predictions—allowed for the prioritization of functionally relevant missense variants. These findings provide a foundation for subsequent structural modeling and molecular dynamics simulations, aimed at elucidating the mechanistic impact of these mutations on BTD function and BD pathogenesis.

### 3.4. Physicochemical Property Analysis

To evaluate whether the prioritized missense mutations affected the overall physicochemical characteristics of the BTD protein, key parameters were calculated for the WT and each of the seven selected mutant forms using the ExPASy ProtParam tool. The analyzed properties included the aliphatic index (AI), instability index (II), isoelectric point (pI), extinction coefficient, and grand average of hydropathicity (GRAVY) (Table 4). The aliphatic index remained unchanged across all variants at 86.37, indicating no alteration in thermostability. The instability index ranged from 31.41 to 32.97, slightly varying between mutants but remaining below the threshold of 40, suggesting all forms of the protein are predicted to be stable. The isoelectric point (pI) exhibited only minor shifts, falling within a narrow range of 5.75 to 5.90, indicating that mutations did not significantly impact the protein’s net charge at physiological pH. The extinction coefficient, used to estimate protein concentration from absorbance, showed minor variations across variants (74,260 to 75,750), reflecting subtle differences in aromatic residue content. Finally, the GRAVY index, a measure of hydrophobicity, ranged from −0.019 to −0.039, suggesting minimal changes in the overall solubility and hydrophilic nature of the protein. Collectively, these findings indicate that the selected missense mutations do not induce significant alterations in the global physicochemical properties of the BTD protein. These subtle differences support the hypothesis that the pathogenic effects of the mutations are more likely due to local structural perturbations or residue-specific interactions, rather than changes in bulk protein characteristics.

### 3.5. Secondary Structure Architecture and Catalytic Site Localization in BTD

**Figure 3:**
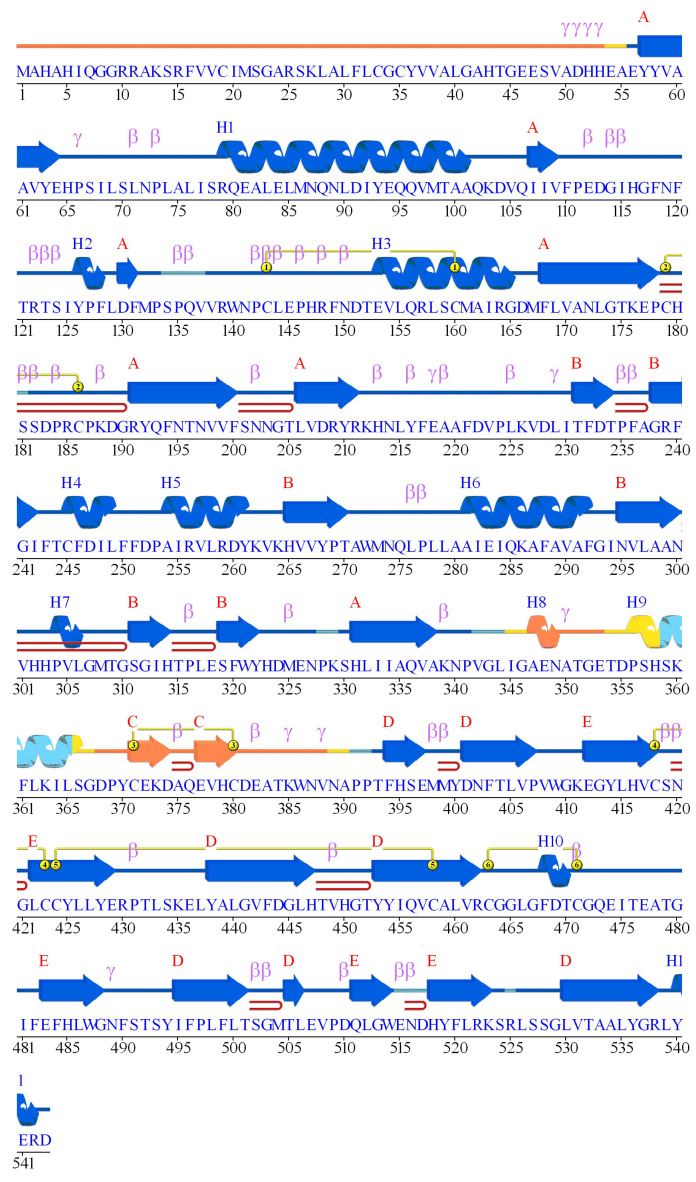
Annotated secondary structure of BTD. The amino acid sequence of BTD is annotated with its predicted secondary structure elements. Blue cylinders represent α-helices (H1–H10), while blue arrows indicate β-strands (labeled A–E), corresponding to β-sheet elements in the protein fold. Turns and coils are represented by loops and ribbons, with β-turns (β), γ-turns (γ), and 310-helices labeled in purple. The catalytic triad residues—Glu112, Lys212, and Cys245, highlighting their spatial distribution within the structural core. This comprehensive annotation provides insights into the folding topology, domain architecture, and potential functional sites of the enzyme.

The secondary structure architecture of the BTD protein was examined to understand the spatial arrangement of structural elements and the localization of catalytic residues. The BTD structure exhibits a well-organized fold composed of alternating α-helices and β-sheets, which are essential for maintaining its overall three-dimensional stability and enzymatic function (Figure 3). The N-terminal region of BTD begins with a β-sheet, followed by an extended α-helix (~20 residues) that contributes to the core scaffold of the protein. This alternating pattern of α-helices and β-sheets continues throughout the structure, creating a stable framework that supports substrate binding and enzymatic catalysis. The three catalytic residues—E112, K212, and C245—are all localized within domain A, corresponding to the conserved nitrilase/amidase homologous domain. Among them, E112 and K212 are situated in random coil regions, which may provide the necessary conformational flexibility for catalytic activity. The third catalytic residue, C245, is embedded within α-helix 4, indicating its structural anchoring within the active site. This configuration suggests that the catalytic triad is strategically positioned, with flexible regions enabling dynamic interactions during catalysis and the helical region providing structural support. The surrounding β-sheet and α-helix elements are likely to stabilize the active site, facilitating efficient substrate recognition and turnover. Together, these observations underscore the crucial role of secondary structure in shaping the active site architecture and catalytic functionality of BTD and emphasize the importance of structural integrity for enzyme performance.

The structural assessment and MD simulations were focused on mutations with higher allele frequency and stronger clinical evidence of pathogenicity. Consequently, they were not prioritized for structural analysis and MD simulations, which focused on variants with higher clinical relevance and potential impact on the protein’s structure and function.

### 3.6. Variant Prioritization for Structural Modeling and Molecular Dynamics Analysis

For detailed structural evaluation, seven missense variants were selected based on their classification as pathogenic or likely pathogenic in ClinVar and their relatively high allele frequency in the Qatari population. These variants were prioritized due to their strong clinical relevance and potential impact on BTD structure and function. As a result, only these selected variants were subjected to *in silico* structural modeling and molecular dynamics (MD) simulations to explore their effects on protein conformation, stability, and active site integrity. Variants with conflicting pathogenicity annotations or extremely low population frequency, despite being protein altering, were excluded from downstream structural analysis, as they lack sufficient clinical or epidemiological support for prioritization. This strategic selection ensured that computational resources were focused on the most biologically and clinically informative mutations.

### 3.7. Quality Assessment of Native Protein and Single Mutants

To study the impact of single mutants at the structural level, we retrieved the BTD structure from the Alphafold program. We introduced single-point mutation in the WT structure of BTD using the PyMoL program. The overall ERRAT quality factor value, expressed as the percentage of the native protein for which the calculated value falls below the 95% rejection limit, was 92.02% (Figure S1a). The z-scores computed by the ProSA-web server for WT and mutants range from −8.65 to −8.80, indicating good-quality models (Table 5). These scores are systematically mapped onto the 3D structure of the protein using a color-coding scheme that visually shows the reliability of different model regions. The resulting plots for the WT and mutant show structural integrity and no potential issues, such as an instability-prone regions in the modeled structure (Figure S1b,c). The overall ERRAT quality factor value for single mutants observed was in the range of 88.0–92.3 (Figure S2). High-resolution structures generally produce 95% or higher [102]. PROCHECK assessed the quality of WT and single mutants of BTD [103,104]. Verifying the results using different tools invariably indicated an excellent quality of the proposed models (both WT and single mutants) (Table 5).

**Figure 4:**
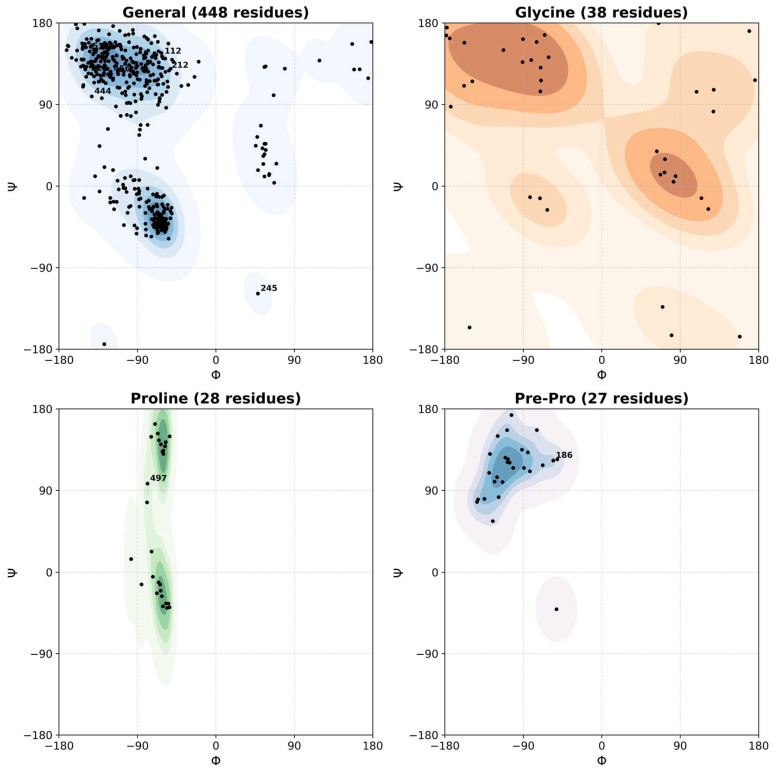
Structural validation and backbone dihedral angle analysis of the BTD-WT. Ramachandran plots illustrating the φ (phi) and ψ (psi) backbone dihedral angle distributions across residue classes: general (non-Gly/Pro), glycine, proline, and pre-proline. Contour density overlays, generated using kernel density estimation, represent favored conformational regions for each class. Black dots denote individual residue angles, with functionally important positions annotated. Catalytic triad residues E112, K212, and C245 are labeled in the general category. Disease-associated mutations C186, E209, H323, D444, Q456, P497, and Q511 are also highlighted in their respective categories to visualize their conformational positioning relative to sterically allowed regions.

To assess the stereochemical quality of the BTD structural models, Ramachandran plots were generated for each residue class: general, glycine, proline, and pre-proline. The majority of residues were observed to cluster within energetically favorable regions, consistent with a well-folded and structurally plausible protein model. Specifically, the catalytic triad residues E112, K212, and C245 were located within allowed conformational regions, supporting the structural integrity of the modeled active site. Additionally, disease-associated mutation sites—including C186, E209, H323, D444, Q456, P497, and Q511—also exhibited φ/ψ angles within favored regions, suggesting that these substitutions are unlikely to significantly disrupt local backbone geometry. Ramachandran plot analysis of the final refined model (Figure 4 and Figure S3) showed that 84.0% of residues were in the most favored regions, 13.9% in additionally allowed regions, 1.0% in generously allowed regions, and only 1.1% in disallowed regions, consistent with high stereochemical quality across both WT and mutant structures [104]. Collectively, these validation results indicated that the 3D models of WT and mutant structures are reliable and can be interpreted with high confidence.

### 3.8. Domain Organization and Mutant Localization in BTD

**Figure 5:**
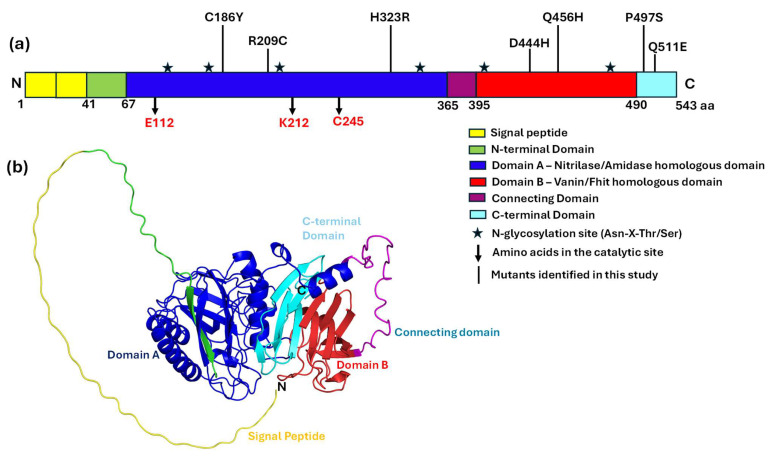
Schematic representation of BTD domains and mutations identified in this study. (**a**) Linear schematic representation of the BTD, showing its structural domains, catalytic residues, glycosylation sites, and identified missense variants. *BTD* consists of two primary domains: domain A (nitrilase/amidase homologous domain, residues 67–365), which contains the catalytic triad (E112, K212, C245), and domain B (vanin/fhit homologous domain, residues 395–490), which is proposed to house the biotin-binding site. The connecting domain (residues 366–394) links these two regions. The locations of six potential N-glycosylation sites (★) and seven missense variants, including the high-frequency variant D444H, are marked. (**b**) Three-dimensional cartoon representation of *BTD* structural domains. The N-terminal region (residues 1–67) includes a signal peptide (yellow, residues 1–41) and the N-terminal domain (green, residues 42–67). Domain A (blue, residues 67–365) contains the catalytic triad, while domain B (red, residues 395–490) harbors biotin-binding residues. The flexible connecting domain (magenta, residues 366–394) links the two main domains. The C-terminal domain (cyan, residues 491–523) contains additional missense variants (P497S, Q511E).

The domain architecture and spatial localization of key mutations within the BTD protein reveal a well-defined structural organization that underpins its enzymatic function. The protein comprises 543 amino acids and features several distinct structural domains, each contributing to its biochemical role. It begins with a signal peptide (residues 1–41), followed by an N-terminal domain (residues 41–67). The primary functional unit, domain A (residues 67–365), shares homology with the nitrilase/amidase family and contains the catalytic triad residues E112, K212, and C245, which are essential for activity (Figure 5a). Domain B (residues 395–490) corresponds to the Vanin/Fhit homologous domain, suggesting a role in substrate binding and regulatory interactions. A connecting domain (residues 365–395) and a C-terminal domain (residues 490–543) further reinforce structural stability. Seven missense variants (C186Y, R209C, H323R, D444H, Q456H, P497S, Q511E) identified in this study map across domains A and B, highlighting that substitutions in either region may compromise protein’s stability or enzymatic function. Within domain A, the catalytic triad (E112, K212, C245) is positioned near a nucleophile elbow formed by β8 and α3, supporting catalytic efficiency. In addition, several N-glycosylation motifs (Asn-X-Thr/Ser) are present, suggesting possible post-translational modifications that may modulate protein stability and activity (Figure 5a).

The three-dimensional structure of the BTD protein offers a detailed view of its domain architecture, illustrating the spatial arrangement of catalytic residues and disease-associated variants within the tertiary fold (Figure 5b). This visualization clearly defines the positioning of domains A and B, along with the connecting and C-terminal domains, and provides insight into how specific mutations may influence biochemical properties. The 3D model also reveals a deeper understanding of the interaction networks within the protein, demonstrating how alterations in specific residues could disrupt structural stability, catalytic efficiency, or substrate binding. This spatial mapping of domains and mutations enhances the understanding of the potential structural and molecular mechanisms underlying BD.

### 3.9. Structural Organization and Catalytic Site Topology of BTD

BTD displays a well-defined secondary structure composed of α-helices, β-sheets, and interconnecting loop regions arranged in a balanced manner. Structural analysis highlights nine α-helices (α1–α9, shown in red) and twenty two β-sheets (β1–β22, shown in yellow), which together provide stability and support the protein’s functional conformation. The nitrilase/amidase homologous domain (domain A), features a strategic distribution of these secondary structural elements, emphasizing their role in maintaining structural integrity and enabling catalytic activity. At the core of domain A lies the catalytic triad, comprising residues E112, K212, and C245, organized around a nucleophile elbow formed by β8 and α3 (Figure S4a,b). This spatial arrangement, characteristic of the nitrilase superfamily, positions E112 within a flexible loop adjacent to β8, while K212 and C245 align near α3 and α5, respectively. Such precise localization supports the catalytic mechanism, where E112 may function as a general base, K212 may stabilize the transition state, and C245 may act as a nucleophile during the hydrolytic process. The close proximity of these residues within the active site highlights their efficiency in substrate binding and catalysis. Overall, the visualization of the catalytic triad within domain A’s secondary structure provides a detailed view of the active site’s topology and offers valuable insights into the enzymatic mechanism of BTD.

### 3.10. Structural Analysis of Missense Mutations in BTD

Structural mapping of the seven prioritized missense variants (C186Y, R209C, H323R, D444H, Q456H, P497S, Q511E) revealed their localization within conserved domains essential for BTD function. These mutations cluster within two structurally and functionally critical regions: (1) the N-terminal catalytic core (domain A, residues ~67–365), which contains the nitrilase-like fold and catalytic triad (E112, K212, C245), and (2) the C-terminal substrate-binding domain (domain B, residues ~395–490), which contributes to the biotin-binding pocket and overall structural stabilization. Within domain A, C186Y, R209C, and H323R are positioned near or within the enzyme’s catalytic machinery. C186 lies in the hydrophobic core of domain A and is highly conserved across species. Replacement with a bulkier tyrosine likely disrupts hydrophobic packing and and destabilizes the domain fold. R209C is located adjacent to the conserved Y-R-K210–212 motif, which stabilizes catalytic Lys212. Substituting of arginine with a cysteine removes a key positive charge, introduces an unpaired thiol group, and may promote aberrant disulfide bond formation or altered loop flexibility. H323R, near the C-terminal boundary of domain A and close to the interface with domain B, replaces histidine with a positively charged arginine, potentially altering local electrostatics and domain-domain alignment. Collectively, these variants are predicted to compromise catalytic triad geometry and weaken the structural framework required for enzymatic hydrolysis.

In domain B, D444H and Q456H occur in a conserved surface-exposed region forming part of the substrate-binding cleft. D444, normally maintains the electrostatic environment for biotin coordination. Its substitution with histidine reverses local charge polarity and may disturb substrate interactions or binding pocket geometry. Q456, located in the same structural loop, contributes to hydrogen bonding networks critical for ligand recognition and stability. 

These alterations are likely to reduce substrate affinity and partially compromise enzymatic efficiency, consistent with the clinical association of D444H with partial BD. The remaining two variants, P497S and Q511E, lie in the distal C-terminal region (~491–543), adjacent to domain B. P497 stabilizes a tight loop through the rigidity of its proline ring. Substitution to a flexible serine increases local mobility, weakens inter-domain packing, and destabilize the structural core. Q511, located in a downstream helical region, contributes to tertiary structure stability. Substitution with a negatively charged glutamate may disrupt surrounding salt bridges or alter solvent interactions, contributing to local structural rearrangements.

**Figure 6:**
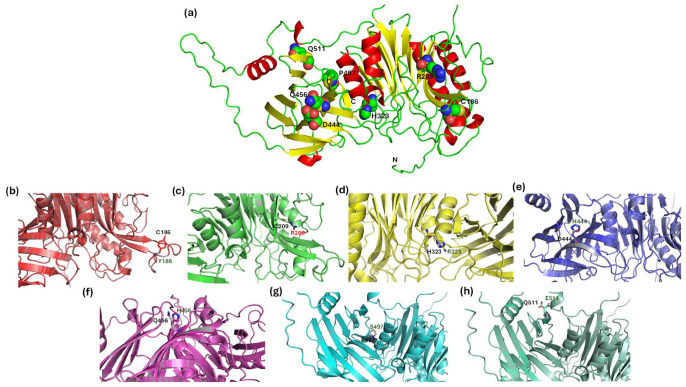
Structural comparison between WT and single mutants of the BTD. (**a**) The three-dimensional structure of the BTD-WT, with key single-mutation residue positions (C186, R209, H323, D444, Q456, P497, and Q511) represented as spheres to illustrate their spatial distribution within the protein. (**b**–**h**) Superpositions of WT and the corresponding single mutants highlighting local conformational changes: C186Y (**b**), R209C (**c**), H323R (**d**), D444H (**e**), Q456H (**f**), P497S (**g**), and Q511E (**h**). In each panel, the affected residues are shown as sticks and labeled in both WT and mutant structures to illustrate the local structural impact of the substitution, which may influence protein stability and/or function.

The tertiary structure of BTD-WT and the spatial distribution of these variants are illustrated in Figure 6a. Mutations are distributed across α-helices (red), β-sheets (yellow), and loops (green), implicating both buried stabilizing residues and surface-exposed interacting regions. Structural superimposition between WT and mutant models (Figure 6b–h) revealed localized perturbations at the mutation sites, including side-chain conformational shifts. These substitutions introduce changes in charge, polarity, and steric bulk that disrupt local hydrogen bonding, salt bridge networks, or hydrophobic clustering. While the overall backbone remains intact, these subtle alterations likely affect enzyme folding, domain assembly, and substrate recognition. Together, this domain-specific mapping and structural characterization highlight how even localized missense mutations can destabilize the enzyme’s architecture or impair active site configuration. These findings underscore the structural vulnerability of the catalytic and substrate-binding domains, which serve as mutational hotspots in BD. Although no global perturbations are observed, the localized disruptions suggest functionally significant effects that warrant further biochemical and functional studies to clarify their role in BD pathogenesis.

### 3.11. Impact of Missense Mutations on Salt Bridge Networks

Salt bridge analysis using the ESBRI server provided valuable insights into the structural stability of the BTD protein in the context of missense mutations. The BTD-WT contained 48 salt bridges, forming a robust electrostatic network that supports protein stability and integrity. Mutants C186Y, H323R, P497S, and Q511E maintained the same number of salt bridges (48), indicating minimal disruption to the electrostatic landscape and suggesting limited impact on overall stability. The R209C and D444H showed reductions to 46 and 47 salt bridges, respectively, indicating modest decreases in stability without directly affecting the enzyme’s active or binding sites (Table S4). In contrast, Q456H mutant exhibited an increase to 50 salt bridges, which may enhance local stability by reinforcing electrostatic interactions. However, an excess of salt bridges can also rigidify the protein, potentially limiting the dynamic flexibility required for optimal enzymatic function, substrate binding, or conformational adaptability. Overall, while these variations did not cause major structural disruptions, they highlight the delicate balance between stability and flexibility essential for proper BTD function. Even modest changes in electrostatic interactions may subtly influence the protein’s structural mechanism.

### 3.12. Electrostatic Potential and Binding Affinity Analysis

Electrostatic properties estimated using generalized Born radii revealed how single-point mutations affect BTD’s electrostatic potential and stability (Figure S5). These changes were further assessed by calculating the total energy of each structure, where more negative values indicate greater stability. The BTD-WT showed a total energy of −60,499 kcal/mol, serving as the baseline for comparison. Mutants H323R, D444H, and P497S exhibited higher (less negative) energies of −60,380, −60,449, and −60,496 kcal/mol, respectively, indicating reduced stability. Notably, D444H lies near the catalytic triad(E112, K212, and C245), where destabilization could impair the orientation and electrostatic environment essential for catalytic activity. Similarly, P497S, located in a flexible loop region, increased electrostatic surface potential, likely disrupting substrate access to the active site. Conversely, C186Y, R209C, Q456H, and Q511E demonstrated lower (more negative) energies, (−60,504 to −61,320 kcal/mol), suggesting increased stability. Among these, Q511E showed the greatest reduction in electrostatic potential, which may contribute to enhanced structural rigidity near substrate-binding regions. However, because these stabilizing effects are distal from the active site, they are unlikely to severely impair enzymatic catalysis. Overall, these findings suggest that mutations affecting residues near or within the active site (e.g., D444H) are more likely to perturb enzymatic function by altering charge distribution and weakening interaction networks surrounding the active residues (E112, K212, and C245). These electrostatic shifts could reduce the enzyme’s efficiency in binding and processing biotin, thereby contributing to disease phenotypes.

### 3.13. Protein Structure Conformational Flexibility and Stability Analyses

**Figure 7:**
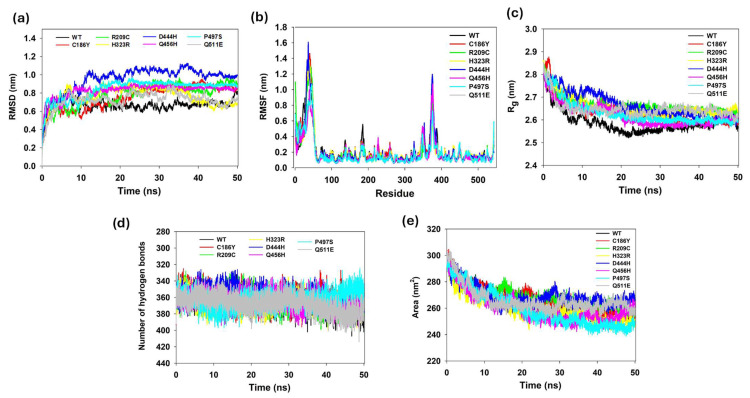
Molecular dynamics simulation analysis of WT and single mutants of BTD. MD simulation trajectory analysis of WT-BTD and its single mutant variants (C186Y, R209C, H323R, D444H, Q456H, P497S, Q511E) over a 50 ns simulation period. (**a**) Root mean square deviation (RMSD) plot showing structural stability fluctuations over time. (**b**) Per-residue root mean square fluctuation (RMSF) analysis highlighting regions of flexibility within the protein structure. (**c**) Radius of gyration (R_g_) indicating changes in protein compactness and structural integrity. (**d**) Hydrogen bond analysis showing the average number of intra-protein hydrogen bonds over the simulation period. (**e**) Solvent-accessible surface area (SASA) plot demonstrating solvent exposure and protein surface dynamics.

MD simulation were performed for 50 ns to assess atomic-level changes in the BTD stability. The structure of BTD consists of single polypeptide chains with 543 residues and a total molecular weight of 61.2 kDa. Simulation trajectories of WT and all seven mutants (C186Y, R209C, H323R, D444H, Q456H, P497S, and Q511E) were analyzed. Root mean square deviation (RMSD) values quantified global stability changes. Average RMSDs 0.66 (WT), 0.75 (C186Y), 0.78 (R209C), 0.76 (H323R), 0.95 (D444H), 0.82 (Q456H), 0.84 (P497S) and 0.72 (Q511E) nms (Figure 7a). All values remained below 3 Å, indicating that the systems were converged. The higher RMSD observed in D444H (~4 Å difference vs. WT) indicates the most pronounced destabilization among single mutants. To understand the effect of the mutants on the dynamic behavior of the residues, the RMSF of WT and mutant structures was also calculated. WT residues fluctuated within a range of ~0.06–0.8 nm, consistent with stable dynamics (Figure 7b). D444H exhibits the highest local flexibility, with several residues fluctuating by up to ~0.50 nm, indicating significant perturbation in structural stability. Other mutants, including C186Y, R209C, H323R, Q456H, P497S, and Q511E, show RMSF values generally within the ~0.10 to 0.25 nm range, with localized increases in flexibility near the mutation sites (Figure 7b). H323R and Q456H displayed slightly broader fluctuation patterns in loop regions, which may influence conformational adaptability. D444H induces the most prominent deviation from WT, potentially destabilizing the enzyme’s structural integrity and functional conformation (Figure 7b). The highest fluctuation peaks (>3 Å) were observed at positions 1–78 across WT and mutants (Figure 7b).

To further validate the above results, WT and mutant proteins were subjected to radius of gyration (R_g_) analysis to measure their compactness. The R_g_ was calculated using the equation where r represents the position of atom I, and ri—RCM is the distance between atom I and the molecule’s center of mass. The R_g_ plot (Figure 7c) indicated that the WT exhibited an average R_g_ value of around 2.66 nm, implying a dense arrangement of the secondary structures (α, β, α + β, α/β) inside the three-dimensional conformation of WT BTD (Figure 7c). Mutant D444H exhibited the highest R_g_ value of ~2.66 nm, followed by the R209H mutant (~2.66 nm). The mutants H323R and Q511 exhibited a lesser R_g_ value of 2.65 nm, which was less than WT; these results align well with pathogenicity and stability prediction results. Other single mutants (C186Y, P497S, Q456H) showed R_g_ values in the 2.61–2.63 nm range. The results predicted that all seven mutants displayed significant deviation patterns in the RMSD, RMSF, and R_g_ values compared with WT, indicating a loss of compactness.

Several structurally destabilizing mutations, such as C186Y, D444H, Q456H, and P497S, were located within or near critical structural elements of BTD. C186Y and Q456H reside in conserved β-sheet regions of the catalytic domain, suggesting direct effects on enzyme integrity. D444H lies adjacent to the catalytic triad and shows substantial deviations in RMSD and radius of gyration, indicating local destabilization. P497S, located in a loop region of the C-terminal domain, was associated with increased flexibility and loss of salt bridges, supporting its destabilizing impact.

Furthermore, although none of the missense variants directly affect the catalytic triad residues E112, K212, or C245, several destabilizing mutations, such as R209C, D444H, and Q456H, are located in close spatial proximity or within the dynamic interaction network of these active site residues. Electrostatic surface mapping and salt bridge analysis revealed that these mutations perturb the charge distribution around domain A, which harbors the catalytic core. Variants such as R209C remove a positively charged arginine adjacent to the YRK210–212 motif that stabilizes K212, while D444H introduces a histidine that alters the local electrostatic field and weakens long-range interactions. These changes were accompanied by increased RMSD, RMSF, and Rg values, indicating a loss of structural compactness and greater conformational flexibility near the active pocket. Given that E112 functions as a general acid/base, K212 stabilizes the transition state, and C245 acts as the nucleophile in the hydrolysis mechanism, even subtle shifts in positioning or local electrostatics can impair substrate recognition and catalytic turnover. Collectively, these results confirm that electrostatic and dynamic changes near E112, K212, and C245 correlate strongly with the enzymatic function of BTD, providing a mechanistic basis for the observed pathogenicity of these variants.

### 3.14. Hydrogen Bond Analysis of WT and Single Mutants

To assess the influence of point mutations on structural stability, the average number of intramolecular hydrogen bonds was computed from 50 ns MD trajectories (Figure 7d). The WT-BTD maintained a robust hydrogen bonding network with an average of 366 hydrogen bonds. Among the variants, Q511E exhibited the highest average of 367, suggesting minimal structural disruption or enhanced stabilization (Figure 7d). Mutants H323R (365) and R209C (364) also closely resembled WT levels. Q456H (364) and R209C (364) showed slightly reduced bonding, while C186Y (361) and P497S (362) indicated further decline. D444H had the lowest average (358), suggesting impaired hydrogen bonding and potential destabilization (Figure 7d). The descending order of hydrogen bond counts was: Q511E > WT > H323R > R209C > Q456H > P497S > C186Y > D444H. These findings highlight the mutation-specific impact on hydrogen bond networks and suggest that D444H and C186Y may impair structural stability more severely than other variants.

### 3.15. Solvent Accessibility and Surface Dynamics

To assess the influence of missense mutations on the surface exposure and structural compactness of BTD, solvent accessible surface area (SASA) was calculated from 50 ns MD trajectories. The WT-BTD exhibited an average SASA of 264.27 nm^2^, indicative of a moderately solvent-exposed and dynamically stable conformation. Among the mutant proteins, D444H displayed the highest SASA (269.11 nm^2^), followed closely by R209C (268.32 nm^2^) and C186Y (267.74 nm^2^), suggesting that these substitutions may increase surface exposure and possibly promote localized unfolding or enhanced flexibility. In contrast, H323R (259.67 nm^2^), P497S (259.36 nm^2^), and Q456H (260.20 nm^2^) showed reduced SASA values relative to WT, indicating a tendency toward a more compact and potentially rigid structure. The Q511E mutant exhibited a SASA of 266.03 nm^2^, marginally higher than WT, implying only subtle alterations in surface dynamics. These findings demonstrate that specific mutations, particularly D444H and R209C, can significantly alter the solvent-accessible surface landscape of BTD, potentially impacting its conformational plasticity and functional stability (Figure 7e).

### 3.16. Principal Component Analysis (PCA) of Conformational Dynamics

**Figure 8:**
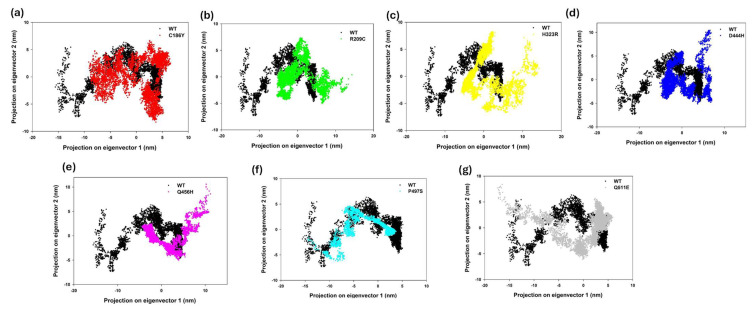
Principal component analysis (PCA) was performed to evaluate the conformational dynamics of WT and single mutants of BTD over the 50 ns molecular dynamics simulation. The two-dimensional projections of the first two principal components (PC1 and PC2) are shown, with WT structures represented in black and mutants in distinct colors. (**a**) C186Y mutant (red) shows broader conformational sampling compared to WT. (**b**) R209C mutant (green) reveals altered cluster distribution. (**c**) H323R mutant (yellow) displays distinct separation from WT dynamics. (**d**) D444H mutant (blue) indicates increased deviation from WT conformations. (**e**) Q456H mutant (magenta) exhibits an expanded conformational space. (**f**) P497S mutant (cyan) shows moderate shifts in structural sampling. (**g**) Q511E mutant (gray) demonstrates altered conformational dynamics compared to WT. Overall, the observed distributions illustrate how single amino acid substitutions modulate the conformational flexibility and global motion of BTD.

To evaluate the global conformational changes induced by single-point mutations in the BTD protein, principal component analysis (PCA) was performed using the Cα atomic coordinates from 50 ns molecular dynamics trajectories. The projections of each trajectory onto the first two principal components (PC1 and PC2), which represent the dominant collective motions, are as shown in Figure 8. Superimposition of all seven mutants with WT on the PC1–PC2 projection space reveals varying degrees of conformational overlap, with Q511E clustering most closely to WT, while D444H and H323R diverge significantly, reflecting mutation-specific shifts in essential dynamics (Figure S6). The WT structure (black) exhibited a compact and confined distribution, indicative of stable conformational dynamics. In contrast, mutant variants such as D444H (blue), Q456H (magenta), and P497S (cyan) occupied broader and more dispersed regions of the phase space, suggesting increased structural flexibility and greater sampling of alternative conformations. The spread of these clusters implies perturbed backbone mobility and possible disruption of structural integrity relative to the WT. To further dissect mutation-specific impacts, pairwise comparisons of WT with each mutant were visualized (Figure 8a–g). Mutants C186Y (red) and R209C (green) showed relatively moderate deviations, with their trajectories partially overlapping the WT ensemble (Figure 8a,b), indicating retention of native-like motion. The H323R mutant (yellow) demonstrated the most pronounced divergence, occupying a distinct conformational basin with minimal overlap with WT (Figure 8c). This result suggests a substantial shift in the global motion profile that may compromise structural or functional domains. Similarly, D444H exhibited a highly dispersed and shifted trajectory cloud (Figure 8d), indicative of enhanced flexibility and reduced structural coherence. Q456H and P497S (Figure 8e,f) also deviated significantly from WT, with the former displaying an expanded motion range that may affect domain stability. In contrast, Q511E (gray) showed the least deviation, maintaining a tightly clustered distribution closely overlapping the WT conformational space, consistent with minimal perturbation to the native fold (Figure 8g). These analyses reveal that while certain mutations (e.g., Q511E) exert minimal structural disruption, others (H323R, D444H, and Q456H) induce pronounced alterations in the protein’s essential dynamics. These changes in the conformational landscape may impair enzymatic efficiency by destabilizing key structural motifs or interfering with allosteric transitions critical for substrate binding and catalysis.

### 3.17. Covariance Matrix Analysis of Residue Correlations in WT and Mutant BTDs

To investigate the effect of missense mutations on internal protein dynamics, dynamic cross-correlation matrices were computed using Cα atoms from molecular dynamics trajectories (Figure S7). The wild-type (WT) BTD exhibited a balanced distribution of positively and negatively correlated motions, reflecting stable and cooperative residue–residue interactions essential for enzymatic function (Figure S7a). Among the mutants, C186Y and R209C displayed moderate loss of coordinated dynamics, with mild increases in anti-correlated regions (Figure S7b,c). H323R and D444H demonstrated pronounced deviations, with enhanced anti-correlated motions (min D444H: −0.477 nm^2^), particularly near the catalytic core, suggesting potential destabilization (Figure S7d,e). Q456H and P497S showed dispersed and weakened correlations, indicating impaired structural communication (Figure S7f,g). Q511E most closely resembled WT, maintaining strong long-range correlations and preserved motion across structural domains (max: +2.11 nm^2^) (Figure S7h). Based on the Cα DCCM patterns, Q511E exhibited the highest dynamic similarity to the WT structure, followed by C186Y, R209C, Q456H, P497S, H323R, and D444H, indicating a progressive deviation in correlated motions across the mutant proteins. These results highlight how specific variants disrupt the global dynamic network of BTD, potentially impairing its structural stability and catalytic efficiency.

### 3.18. Free Energy Landscape of WT and Mutant BTDs

**Figure 9:**
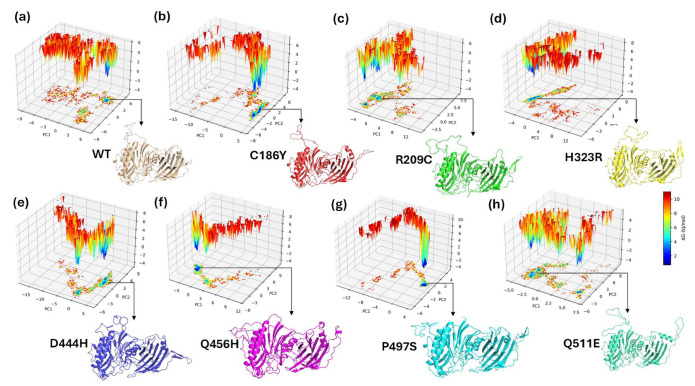
Free energy landscape (FEL) of WT and single mutants of BTD. The Gibbs free energy landscapes were generated using the first two principal components (PC1 and PC2) from principal component analysis (PCA) of molecular dynamics trajectories for the apo form of BTD. Panels represent: (**a**) WT, (**b**) C186Y, (**c**) R209C, (**d**) H323R, (**e**) D444H, (**f**) Q456H, (**g**) P497S, and (**h**) Q511E variants. The FEL plots depict conformational distributions and corresponding free energy values (ΔG, in kJ/mol), with a color gradient from blue (low-energy, stable conformations) to red (high-energy, less stable conformations). Representative protein structures from the lowest-energy basins are shown below each plot.

To evaluate the conformational stability and dynamic behavior of WT and single-point mutants of BTD, Gibbs free energy landscapes (FELs) were constructed based on the first two principal components (PC1 and PC2) from the PCA of molecular dynamics simulations (Figure 9a–h). Each 3D FEL plot visualizes the conformational space sampled by the protein during the simulation, with energy minima corresponding to stable states. The color scale represents relative free energy (ΔG in kJ/mol), ranging from low-energy (blue) to high-energy (red) conformations. The WT-BTD exhibited a relatively broad and continuous energy basin, indicating high conformational flexibility and access to multiple low-energy states (Figure 9a). In contrast, several mutants showed distinct deviations in their energy topographies. The C186Y and R209C mutants exhibited more confined basins and sharper energy minima, suggesting restricted structural dynamics and possible local destabilization (Figure 9b,c). The H323R variant revealed a shifted energy minimum along PC1, highlighting a deviation in the dominant motion compared to WT (Figure 9d). D444H displayed multiple deep energy wells, implying the presence of alternative stable conformations and potential conformational heterogeneity (Figure 9e). The Q456H and P497S mutants exhibited narrow, well-defined basins, indicating limited conformational sampling and possible rigidity (Figure 9f,g). The Q511E variant, while distinct from WT, retained a relatively extended energy landscape with lower free energy barriers, suggesting a milder structural impact (Figure 9h). These results demonstrate that specific mutations, especially R209C, H323R, and D444H, induce substantial changes in the energy landscape of BTD, consistent with altered conformational dynamics and potential functional impairment. The FEL analysis reinforces predictions of mutation-induced destabilization and provides structural insight into their likely pathogenic effects.

### 3.19. Clinical, Biochemical, and Genetic Findings of BTD Variants in Middle Eastern Countries

The analysis of clinical and genetic data from Middle Eastern and Turkish patients with BD provided valuable insights into genotype–phenotype correlations across diverse populations (Table 6). In the United Arab Emirates (UAE), a total of 77 BTD cases were reported, with 55/77 (71.4%) presenting symptomatic manifestations, while 22/77 (28.6%) remained asymptomatic. Clinical presentations included global developmental delay, seizures, speech delay, hyperactivity, eczema, and optic atrophy. The most frequent mutations identified were p.D444H, p.C186Y, p.F403V, p.H323R, and p.P497S, with p.D444H being the most prevalent (Table 6).

p.D446H was associated with juvenile optic atrophy, suggesting its potential link to severe ocular manifestations. The high proportion of symptomatic cases highlights the need for effective early screening and targeted interventions to reduce disease burden in the UAE population. In Saudi Arabia, 34 BTD cases were documented, with 31/34 (91.2%) exhibiting severe clinical phenotypes, including seizures, developmental delay, severe acidosis, optic atrophy, hypotonia, and dysarthria. Only 3/34 (8.8%) of cases were asymptomatic, indicating a high clinical burden of pathogenic BTD variants in this population. The predominance of severe manifestations underscores the critical need for robust newborn screening (NBS) and genetic testing protocols to facilitate early detection and management of BTD in Saudi Arabia. The Qatari cohort revealed 14 BTD cases, with 2/14 (14.3%) identified as asymptomatic through newborn screening (NBS). This finding emphasizes the effectiveness of NBS in early diagnosis, which could prevent severe clinical outcomes through timely biotin supplementation and therapeutic interventions. These results support the integration of BTD variants into the national genomic screening program to improve clinical outcomes for affected individuals in Qatar (Table 6). In Lebanon, there was a single BD case (1/1; 100%) presenting with severe symptoms, including seizures, brain atrophy, hyperreflexia, and optic disc pallor, highlighting the potential severity of untreated BD. This case reinforces the importance of early diagnostic strategies and the potential benefit of implementing NBS programs in Lebanon to mitigate severe disease manifestations. In Somalia, there were 33 BD cases reported, of which 26/33 (78.8%) had unreported clinical presentations, while 7/33 (21.2%) exhibited notable mutations including p.P497S, p.D444H, p.P142T, and p.A478P. The p.D444H variant was frequently detected in compound heterozygous states, suggesting its influence on disease severity. The high prevalence of this mutation points to the need for targeted genetic studies and enhanced clinical awareness to improve the diagnosis and management of BTD in Somali populations. In Türkiye, 31 BD cases were reported, displaying a broad clinical spectrum. Of these, 13/31 (41.9%) were symptomatic, showing seizures, hypotonia, vision and hearing impairments, metabolic acidosis, and ataxia, and 18/31 (58.1%) were identified through NBS, indicating the significant impact of early screening in reducing symptomatic presentations. Significant mutations such as p.Q456H, p.R79C, p.Y57Ter, and various frameshift variants were associated with severe biochemical and neurological phenotypes, underlining the necessity for continued genetic screening and follow-up in Türkiye. Overall, p.D444H emerged as a critical variant across multiple Middle Eastern populations, correlating with both mild and severe clinical outcomes depending on zygosity and mutation interaction (Table 6). These findings advocate for the incorporation of specific BTD variants into regional neonatal genomic screening programs to enhance early detection and enable personalized therapeutic strategies, ultimately improving clinical outcomes in these high-risk populations.

## 4. Discussion

Large genomic datasets and population studies have revealed considerable heterogeneity in BTD mutations, with more than 165 distinct variants identified globally [57]. This diversity is especially pronounced in consanguineous populations, where regional founder effects and recurrent homozygosity contribute to increased disease prevalence [11,116]. Populations with high consanguinity rates, particularly in the Middle East, demonstrate a higher prevalence of homozygous and heterozygous mutations in the BTD gene, offering valuable insights into how these genetic variations influence enzyme activity and disease susceptibility [19,20,21,22,23,25,26,27,28,29,30,31,32,33,34,117]. High consanguinity leads to characteristic genotype patterns, often resulting in affected individuals being homozygous for the same mutation, which amplifies disease prevalence and leads to multiple cases within extended families [11,116]. Our study confirms this trend, revealing a notable BTD carrier frequency of 1 in 20 in the Qatari population—comparable to frequencies reported in Saudi Arabia, Türkiye, and the UAE. This is largely driven by the D444H (rs13078881) variant, a known marker of partial BD, which frequently occurs in compound heterozygous states with severe alleles like P497S or H323R.

From the Qatar Genome Program (QGP) cohort (n = 14,669), we identified 21 potentially pathogenic BTD variants—19 SNPs and 2 structural variants (SVs). Of the 14 SNPs amenable to *in silico* modeling, several involved highly conserved residues (e.g., C186, R209, Q456, P497) that are essential for structural stability and catalytic activity. These variants, particularly C186Y and R209C, are predicted to destabilize the enzyme and are consistent with those previously linked to profound BD [18,115,118,119]. In contrast, mutations in moderately conserved residues such as H323 and D444 may result in milder structural disruptions. Four truncating mutations (G34fs, R164fs, Y57Ter, and Q156Ter) were also detected and are predicted to yield non-functional proteins due to premature termination or frameshifts, although experimental validation is still lacking. Two of these variants (rs765906887 and rs397514365) were also classified as SVs, reinforcing their potential clinical relevance. Due to the limitations of current computational tools, which are optimized for analyzing missense mutations, these variants were excluded from *in silico* modeling. However, their presence in the QGP dataset, particularly in a high-consanguinity context, highlights their potential relevance and underscores the need for future experimental validation. Specific missense mutations, such as A478T, D543G, and T479M, were noted for their potential to disrupt the structural core of the enzyme or interfere with surface interactions, highlighting the diverse mechanisms through which genetic variants can influence disease severity [11,55].

Protein stability predictions revealed that 78.6% of the evaluated nonsynonymous SNPs are destabilizing (negative ΔΔG values), supporting enzyme destabilization as a key pathogenic mechanism in BD. Variants such as L535V, A478T, P497S, R209C, and C186Y exhibited significant destabilizing effects, correlating with more severe phenotypes [115]. Conversely, Q511E—a mutation located outside the catalytic core—showed a modest impact on stability and was associated with milder structural deviation, suggesting partial retention of enzymatic function. Interestingly, some variants with positive ΔΔG values (e.g., H323R, Q511E) may still affect enzyme function via misfolding or active site alterations rather than global instability [120]. Physicochemical analyses further revealed that variants like R209C and T532M have high instability indices, while H323R and D444H altered the isoelectric point (pI), potentially disrupting protein interactions and substrate binding [11,55]. These subtle changes, though not globally destabilizing, can still impair function. The high reliability of AlphaFold-based structural models (ERRAT > 88%, ProSA Z-scores) suggests that pathogenicity in BTD likely arises from local conformational disruptions, particularly within catalytic domain A, which includes key residues C186, R209, and H323 [4,121,122].

Molecular dynamics (MD) simulations offer valuable mechanistic insights into how specific missense variants in the BTD gene alter protein stability, conformational flexibility, and enzymatic function. Among the commonly reported pathogenic variants, D444H, H323R, and P497S exhibit distinct destabilization patterns that align with the clinical heterogeneity of BD. The D444H variant, frequently observed in individuals with partial BD, showed increased structural flexibility and conformational heterogeneity. MD simulations revealed elevated root mean square deviation (RMSD) and root mean square fluctuation (RMSF) values, a slight expansion in radius of gyration (Rg), and a notable reduction in intramolecular hydrogen bonds relative to the WT structure. These changes indicate partial unfolding and moderate destabilization without complete loss of structural integrity. This is consistent with the hypomorphic nature of D444H, which retains residual enzymatic activity and is commonly seen in compound heterozygous contexts [8,55,116].

In contrast, H323R and P497S were associated with more pronounced structural perturbations and potentially more severe phenotypes. The H323R mutation disrupted global motion patterns, as shown by distinct clustering in principal component analysis (PCA) and a shifted minimum in the free energy landscape (FEL), indicating a preference for non-native conformations. P497S, situated in a flexible loop region, disrupted a stabilizing salt bridge and introduced conformational constraints that limited global dynamics. These effects were reflected in increased RMSD values, altered Rg, and a narrower FEL basin, suggesting a more rigid and functionally compromised structure. These observations highlight that different mutations destabilize BTD through diverse mechanisms: enhanced flexibility (D444H), altered domain interactions (H323R), and reduced dynamic adaptability (P497S).

To further connect these structural perturbations with enzymatic function, we examined electrostatic and dynamic changes around the catalytic triad (E112, K212, and C245), which underpins BTD’s amidase activity. C245 acts as the nucleophile, while E112 and K212 facilitate proton transfer and transition-state stabilization. Variants such as R209C, located adjacent to the YRK210–212 motif that stabilizes K212, remove a critical positive charge required for optimal triad orientation. Likewise, D444H redistributes electrostatic potential by replacing a negatively charged aspartate with histidine, weakening the long-range charge network that stabilizes the active pocket. These electrostatic perturbations, combined with the altered backbone flexibility seen in RMSF profiles, may misalign the catalytic residues, impairing nucleophile activation and substrate turnover. A slight reduction in salt bridges observed for R209C and D444H supports this functional impact. Thus, the electrostatic and dynamic shifts identified in our simulations provide a molecular explanation for the reduced catalytic efficiency observed in pathogenic BTD variants. Altogether, these MD-derived insights bridge structural dynamics with enzymatic function and clinical severity. They underscore the value of computational modeling for refining genotype–phenotype correlations in BD and align with prior studies highlighting the importance of conformational dynamics in modulating BTD activity [123].

In concordance with previously characterized regional variants, the findings from our study reinforce the existence of a shared mutation spectrum across the greater Middle Eastern population. Recurrent missense variants such as D444H, H323R, and P497S, which have been widely reported in populations from the UAE, Somalia, and Türkiye, were also identified in the Qatari cohort [17,72,73,74,75,114]. This overlap supports the hypothesis of a common ancestral origin or founder effects driving the regional enrichment of these alleles. Importantly, several of these variants, including C186Y, H323R, T532M, and D444H, have been associated with a broad spectrum of clinical presentations—from asymptomatic or partial BD to profound enzymatic impairment—underscoring the multifactorial nature of genotype–phenotype correlations in BTD (Table 6). Of particular interest, R209C and Q511E were newly identified within the Qatari dataset and have not been previously reported in regional genomic studies. Their emergence suggests potential population-specific variants that may be underrepresented in global databases and emphasizes the necessity for continued regional surveillance and clinical annotation. The identification of these variants expands the mutational repertoire associated with BTD in Middle Eastern populations and highlights the critical need for functional validation studies to assess their biochemical and pathogenic consequences [124].

Taken together, this study highlights the importance of integrating large-scale population genomics with *in silico* structural modeling to elucidate the functional consequences of BTD mutations. By contextualizing the Qatari data within broader regional and global frameworks, we advocate for the inclusion of common variants—particularly D444H—in precision-based newborn screening strategies. Such integration can enable earlier diagnosis, personalized biotin therapy, and improved outcomes for individuals at risk in high-consanguinity populations. 

## 5. Conclusions

This study provides a detailed genomic and structural analysis of BD in a greater Middle Eastern population, leveraging whole-genome sequencing data from the Qatar Genome Program (QGP) and molecular dynamics (MD) simulations. A high carrier frequency (1:20) of BTD-related variants was identified, with rs13078881 (D444H) being the most prevalent. MD simulations revealed structural deviations in several BTD mutants, with D444H exhibiting only mild changes, correlating with its association with milder clinical phenotypes. Our findings highlight the critical need to integrate these pathogenic and likely pathogenic variants into national genomic neonatal screening (gNBS) programs to improve early detection and treatment strategies for BTD. The study underscores the utility of *in silico* tools and MD simulations in elucidating the structural and functional impacts of BTD mutations, providing a molecular basis for genotype–phenotype correlations. Overall, this research enhances our understanding of BD’s genetic landscape in populations with high consanguinity, supporting precision medicine approaches. Future studies should prioritize the experimental validation of identified variants, broaden genomic screening initiatives, and investigate personalized biotin therapy approaches to enhance clinical outcomes [125,126,127,128,129].

## Figures and Tables

**Table 1:** Summary of SNPs and SVs (pathogenic and likely pathogenic) identified in the QGP cohort (n = 14,669) from the BTD gene, including genotype frequencies, homozygous variants, and carrier frequency comparisons with global populations.

Genomic Coordinates	RS ID	Nucleotide Change	Amino Acid Change	Classification (ClinVar)	Genotype	Genotype (GT) n (%)					
						* QGP (n = 14,669)			^&^ IP (n = 2504)		
						Homo	Hetero	WT	Homo	Hetero	WT
3:15644413	rs397514369	BTD: c.557G>A	C186Y	P/LP	AA/AG/GG	0 (0%)	14 (0.09%)	14,655 (99.9%)	0 (0%)	0 (0%)	2504 (100%)
3:15644481	rs369102875	BTD: c.565C>T	R209C	P	TT/TC/CC	1 (0.006%)	7 (0.04%)	14,661 (99.9%)	0 (0%)	0 (0%)	2504 (100%)
3:15644824	rs397507176	BTD: c.968A>G	H323R	LP	GG/GA/AA	2 (0.01%)	49 (0.33%)	14,618 (99.44%)	0 (0%)	24 (0.95%)	2480 (99.04%)
3:15645186	rs13078881	BTD: c.1330G>C	D444H	P	CC/CG/GG	22 (0.14%)	608 (4.14%)	14,039 (95.7%)	4 (0.15%)	85 (3.39%)	2415 (96.44%)
3:15645224	rs80338685	BTD: c.1368A>C	Q456H	P	CC/CA/AA	0 (0%)	10 (0.06%)	14,659 (99.9%)	0 (0%)	1 (0.03%)	2503 (99.9%)
3:15645345	rs138818907	BTD: c.1489C>T	P497S	P	TT/TC/CC	0 (0%)	6 (0.04%)	14,663 (99.9%)	0 (0%)	1 (0.03%)	2503 (99.9%)
3:15645387	rs397514427	BTD: c.1531C>G	Q511E	LP	GG/GC/CC	0 (0%)	2 (0.01%)	14,667 (99.9%)	0 (0%)	0 (0%)	2504 (99.9%)
3:15645288	rs181396238	BTD: c.G>A	A478T	P	AA/AG/GG	0 (0%)	1 (0.006%)	14,668 (99.9%)	0 (0%)	3 (0.11%)	2501 (99.88%)
3:15645292	rs142249642	BTD: c.C>T	T479M	P/LP	TT/TC/CC	0 (0%)	6 (0.04%)	14,663 (99.9%)	0 (0%)	3 (0.11%)	2501 (99.8%)
3:15645451	rs104893688	BTD: c.C>T	T532M	P/LP	TT/TC/CC	0 (0%)	1 (0.006%)	14,668 (99.9%)	0 (0%)	1 (0.03%)	2503 (99.9%)
3:15645484	rs1050035768	BTD: c.A>G	D543G	LP	GG/GA/AA	0 (0%)	2 (0.01%)	14,667 (99.9%)	0 (0%)	0 (0%)	2504 (100%)
3:15635478	rs765906887	BTD: c.CGG>C	G34fs	P	CC/CG/GG	0 (0%)	9 (0.06%)	14,660 (99.9%)	0 (0%)	0 (0%)	2504 (100%)
3:15635550	rs397514339	BTD: c.T>G	Y57Ter	P	GG/GT/TT	0 (0%)	1 (0.006%)	14,668 (99.9%)	0 (0%)	0 (0%)	2504 (100%)
3:15644322	rs397514362	BTD: c.C>T	Q156Ter	P	TT/TC/CC	0 (0%)	2 (0.01%)	14,667 (99.9%)	0 (0%)	0 (0%)	2504 (100%)
3:15644345	rs397514365	BTD: c.CAG>C	R164fs	LP	CC/GC/GG	0 (0%)	3 (0.02%)	14,666 (99.9%)	0 (0%)	0 (0%)	2504 (100%)
3:15641920	rs976185636	BTD: c.A>G	I108V	P ^¥^	GG/GA/AA	0 (0%)	1 (0.006%)	14,668 (99.9%)	0 (0%)	0 (0%)	2504 (100%)
3:15645295	rs558477960	BTD: c.G>A	G480E	P ^¥^	AA/AG/GG	0 (0%)	20 (0.13%)	14,649 (99.8%)	0 (0%)	1 (0.03%)	2503 (99.9%)
3:15645459	rs771537277	BTD: c.C>G	L535V	P ^¥^	GG/GC/CC	0 (0%)	1 (0.006%)	14,668 (99.9%)	0 (0%)	0 (0%)	2504 (100%)
3:15645647	rs530872564	BTD: c.G>A	-	P ^¥^	AA/AG/GG	0 (0%)	1 (0.006%)	14,668 (99.9%)	0 (0%)	2 (0.07%)	2502 (99.9%)
3:15635478	rs765906887	BTD:c.CGG>TGG,C	-	P	TGG:TGG/CGG:TGG/CGG:CGG	0 (0%)	9 (0.06%)	14,660 (99.9%)	0 (0%)	0 (0%)	2504 (100%)
3:15644345	rs397514365	BTD:c.CAG>C	-	LP	C:C/CAG:C/CAG:CAG	0 (0%)	3 (0.02%)	14,666 (99.9%)	0 (0%)	0 (0%)	2504 (100%)

* QGP = Qatar Genome Program from Qatar Precision Health Institute. ^&^ IP = international population (from Ensembl database); P—pathogenic; LP—likely pathogenic; ^¥^ P—conflicting classification of pathogenicity—pathogenic/likely pathogenic/uncertain significance.

**Table 2:** Predictions of pathogenicity of BTD nsSNPs using multiple *in silico* tools.

RS ID	Mutation	^#^ PANTHER Score	^&^ PhD-SNP Score	^$^ SIFT Score	^^^ SNAP Score	^%^ Meta-SNP Score	RI *
rs397514369	C186Y	Disease 0.918	Disease 0.862	Disease 0.010	Disease 0.735	Disease 0.799	6
rs369102875	R209C	Disease 0.979	Disease 0.867	Disease 0.000	Disease 0.720	Disease 0.819	6
rs397507176	H323R	Disease 0.511	Disease 0.318	Neutral 0.270	Disease 0.695	Neutral 0.315	4
rs13078881	D444H	Disease 0.692	Disease 0.793	Disease 0.010	Disease 0.825	Disease 0.811	6
rs80338685	Q456H	Disease 0.820	Disease 0.755	Disease 0.000	Disease 0.810	Disease 0.825	6
rs138818907	P497S	Disease 0.779	Disease 0.643	Disease 0.000	Disease 0.720	Disease 0.697	4
rs397514427	Q511E	Neutral 0.194	Neutral 0.170	Disease 0.800	Neutral 0.125	Neutral 0.156	7
rs976185636	I108V	Neutral 0.331	Neutral 0.485	Disease 0.000	Disease 0.560	Neutral 0.425	2
rs181396238	A478T	Disease 0.606	Neutral 0.410	Disease 0.000	Disease 0.640	Disease 0.535	1
rs142249642	T479M	Neutral 0.357	Neutral 0.167	Neutral 0.190	Neutral 0.180	Neutral 0.212	6
rs558477960	G480E	Neutral 0.319	Neutral 0.258	Disease 0.020	Disease 0.550	Neutral 0.260	5
rs104893688	T532M	Disease 0.686	Disease 0.602	Disease 0.000	Disease 0.740	Disease 0.720	4
rs771537277	L535V	Disease 0.537	Neutral 0.327	Disease 0.000	Disease 0.700	Neutral 0.361	3
rs1050035768	D543G	Neutral 0.330	Neutral 0.295	Disease 0.0000	Disease 0.670	Neutral 0.356	3

Table legend: * RI (reliability index) ranges from 0 to 10, indicating the confidence level of predictions. For ^#^ PANTHER, ^&^ PhD-SNP, ^$^ SIFT, ^^^ SNAP, and ^%^ Meta-SNP, scores range between 0 and 1, where a score > 0.5 predicts a disease-causing mutation. In contrast, ^$^ SIFT scores ≤ 0.05 indicate deleterious mutations, while higher values predict neutral variants. Meta-SNP ranking prioritizes variants based on their predicted pathogenic impact. CADD scores assess mutation severity, with higher values indicating greater pathogenicity. ^%^ Meta-SNP score ranking: Q456H > R209C > D444H > C186Y > T532M > P497S > A478T > I108V > L535V > D543G > H323R > G480E > T479M > Q511.

**Table 3:** Results of instability and function change analysis using I-Mutant, MuPro, and DDGun servers for all single mutants in BTD enzyme causing BD.

BTD Enzyme	I-Mutant 2.0		MuPro		DDGun	
	Stability Effect	ΔΔG (kcal/mol)	Stability Effect	ΔΔG (kcal/mol)	Stability Effect	ΔΔG (kcal/mol)
BTD_C186Y	Decrease	−0.20	Decrease	−1.33	Decrease	−0.3
BTD_R209C	Decrease	−1.63	Decrease	−1.07	Decrease	−1.6
BTD_D444H	Decrease	−0.12	Decrease	−0.92	Decrease	0
BTD_Q456H	Decrease	−0.76	Decrease	−1.14	Decrease	−0.1
BTD_P497S	Decrease	−1.65	Decrease	−0.83	Decrease	−0.5
BTD_L535V	Decrease	−2.91	Decrease	−0.93	Decrease	−0.1
BTD_D543G	Decrease	−1.31	Decrease	−1.94	Decrease	−0.2
BTD_I108V	Decrease	−1.43	Decrease	−0.39	Decrease	−0.4
BTD_H323R	Increase	0.42	Decrease	−0.83	Decrease	−0.2
BTD_G480E	Increase	0.43	Decrease	0.60	Neutral	0
BTD_Q511E	Increase	0.26	Decrease	−0.40	Increase	−0.1
BTD_A478T	Decrease	−2.05	Decrease	−1.03	Neutral	0
BTD_T479M	Decrease	−0.54	Decrease	−0.37	Neutral	0
BTD_T532M	Decrease	−1.08	Increase	0.63	Neutral	0

ΔΔG: ΔG (NewProtein) − ΔG (WT) in Kcal/mol; ΔΔG < 0: decrease stability; ΔΔG > 0: increase stability. Ranking based on I-mutant 2.0: L535V > A478T > P497S > R209C > I108V > D543G > T532M > Q456H > T479M > C186Y > D444H > Q511E > H323R > G480E.

**Table 4:** Physiochemical properties of WT and single mutants in BTD enzyme.

BTD Enzyme	Aliphatic Index	Instability Index	* pI	Extension Coefficient	* GRAVY
BTD_WT	86.37	31.95	5.81	75,260	−0.032
BTD_C186Y		31.68	5.81	75,750	−0.039
BTD_R209C		32.23	5.75	75,260	−0.019
BTD_H323R		31.41	5.83	74,260	−0.034
BTD_D444H		31.53	5.90	74,260	−0.031
BTD_Q456H		32.32	5.84	74,260	−0.031
BTD_P497S		31.60	5.81	74,260	−0.030
BTD_Q511E		31.95	5.76	74,260	−0.032
BTD_A478T		31.95	5.81	75,260	−0.036
BTD_T479M		32.11	5.81	74,260	−0.027
BTD_T532M		32.33	5.81	74,260	−0.027
BTD_D543G		31.79	5.87	74,260	−0.026
BTD_I108V		31.95	5.81	74,260	−0.032
BTD_G480E		32.97	5.76	74,260	−0.037
BTD_L535V		31.81	5.81	74,260	−0.031

* pI—isoelectric point * GRAVY—grand average of hydropathy.

**Table 5:** Quality assessment and reliability of AlphaFold models of WT and single mutants of BTD enzyme.

BTD Enzyme	ERRAT Overall Quality Factor	ProSA-Web (Z-Score) *	Ramachandran Plot		
			Favored Region ^†^	Allowed Region ^†^	Disallowed Region ^†^
BTD_WT	92.02	−8.75	84%	15%	1.1%
BTD_C186Y	88.79	−8.69	84%	15%	1.1%
BTD_R209C	92.24	−8.77	84%	15%	1.1%
BTD_H323R	91.16	−8.72	84%	15%	1.1%
BTD_D444H	92.04	−8.76	84%	15%	1.1%
BTD_Q456H	92.02	−8.79	84%	15%	1.1%
BTD_P497S	92.02	−8.76	83.8%	15.2%	1.1%
BTD_Q511E	92.02	−8.74	84%	15%	1.1%

* Indicative of overall homology model quality using ProSA-web server. ^†^ Based on Ramachandran plot of predicted model by PROCHECK-SAVES server.

**Table 6:** Summary of clinical findings and genotype–phenotype correlation in Arab and Türkiye patients with BD.

Origin	Phenotype	Age of Onset	N	Clinical Presentation	Method Of Diagnosis	Nucleotide Change	Amino Acid Changes	Ref
UAE	BTD	<1 years	7	Not reported	Biochemical tests (NBS) and molecular genetics tests	c.1330G>C ^†^ c.1207T>G c.968A>G c.1489C>T c.557G>A	p.D444H ^†^ p.F403V p.H323R p.P497S p.C186Y	[17]
UAE	BTD	<1.5 years	13	Not reported	Biochemical tests and molecular genetics tests	c.1330G>C c.557G>A	p.D444H p.C186Y	[72]
UAE	BTD	12 years	1	Global developmental delay, seizure, blind sleep disturbance	Biochemical tests and molecular genetics tests	c.560del	p.P187Qfs*77	[75]
		11 years	1	Speech delay, learning difficulty		c.1207T>G c.1330G>C	p.F403V p.D444H	
		8 years	1	Strabismus, delayed speech, hyperactivity, hyperpigmented and hypopigmented macules, hearing loss		c.557G>A c.1330G>C	p.C186Y p.D444H	
		6 years	1	Speech delay, learning difficulty		c.1330G>C	p.D444H	
UAE	BTD	≤3 years	2	Speech delays and eczema	Biochemical tests and molecular genetics tests	c.557G>A c.1330G>C	p.F403V p.D444H	[79]
UAE	BTD	<1 year	2	Not reported	Biochemical tests (NBS) and molecular genetics tests	c.322A>G c.1595C>T	p.I108V p.T532M	[73]
UAE	BTD	<1 year	14 ^‡^	Global developmental delay, seizure, blindness, sleep disturbance, hearing impairment	Biochemical tests and molecular genetics tests	c.476G> c.1330G>C c.1595C>T c.968A>G c.1207T>G c.557G>A c.1489C>T c.257T>C	p.S159N p.D444H p.T532M p.H323R p.F403V p.C186Y p.P497S p.M86T	[74]
			34 ^§^			c.626G>A c.1368A>C c.380C>T c.1420G>T c.470G>A c.557G>A c.1330G>C c.968A>G c.476G>A c.1595C>T c.424C>A c.476G>A c.922A>C c.1489C>T	p.R209H p.Q456H p.P127L p.E474X p.R157H p.C186Y p.D444H p.H323R p.S159N p.T532M p.P142T p.S159D p.M308L p.P497S	
UAE	BTD	13 years	1	Juvenile optic atrophy, functional visual loss, bilateral optic nerve head pallor, mild thinning of the orbital segments of the optic nerves, and hearing difficulty	Biochemical tests and molecular genetics tests	c.1213T.G c.1336G.C	p.F405V p.D446H	[76]
Saudi Arabia	BTD	<1 year	1	Degenerative brain disease, convulsive disorder, and seborrheic dermatitis	Biochemical tests (NBS)	Not reported	Not reported	[80]
			20	Seizures, dermatitis, hypotonia, poor feeding, sensorineural deafness, poor speech and optic atrophy				[16]
			3	Not reported				[78]
		≤17 years	10	Severe acidosis, seizures, coma, global developmental delay, generalized weakness, dysphasia, loss of hearing and vision, severe spastic quadriparesis, ophthalmoplegia, dysarthria, convulsions (generalized tonic/clonic), ataxia, lethargy, ataxia, slurred speech, severe hypotonia, hyporeflexia and choreoathetosis movements				[77]
Qatar	BTD	<1 year	2	Not reported	Biochemical tests (NBS)	Not reported	Not reported	[71]
			12					[25]
Lebanon	BTD	<1 year	1	Seizure; brain atrophy, global developmental delay, hyperreflexia, optic disc pallor, large for gestational age	Biochemical tests	Not reported	Not reported	[114]
Somalia	BTD	<1 year	26/33	Not reported	Biochemical tests and molecular genetics tests	c.424C>A c.1489C>T c.1284C>T c.1432G>C	p.P142T p.P497S p.Y428Y p.A478P	[115]
			7/33				p.H65R, p.D444H ^‡‡^ 98:d7i3, p.D444H ^‡‡^ p.A171T, p.D444H ^‡‡^ p.V417F, p.D444H ^‡‡^ p.Y210C, p.D444H ^‡‡^ p.R538H	
Türkiye	BTD	<1.5 years (symptomatic)	13	Seizures, hypotonia; lethargy; mental retardation, ataxia, vision impairment, hearing impairment alopecia, rash, conjunctivitis Respiratory problems, GI, gastrointestinal, metabolic acidosis, hyperammonaemia, organic aciduria (elevated concentrations of 3 hydroxyisovalerate, with and without methylcitrate, 3-hydroxypropionate, 3-methylcrotonylglycine, and/or lactate)	Biochemical tests and molecular genetics tests Biochemical tests and molecular genetics tests	c.171T>G c.98G:del7ins3 c.587C>G c.235C>T c.612C>T c.1368A>C c.100G>A c.1369G>A	p.Y57Ter Frameshift p.T196R p.R79C p.R538C p.Q456H 3′Splice site p.V457M	[18]
		<1.5 years ascertained by newborn screening (NBS)	18	Not Reported		c.235C>T c.470G>A c.1595C>T c.557G>A c.98G:del7ins3 c.100G>A c.1330G>C, c.511G>A c.929G>A c.1368A>C	p.R79C p.R157H p.T532M p.C186Y Frameshift 3′Splice site p.D444H, p.A171T G310E Q456H	

Data have been taken from The Centre for Arab Genomic Studies (CAGS) (accessed on 02/07/2024) ^†^ Mutations have been found in more proband/siblings. ^‡^ Mutations detected in Emirati citizens. § Mutations detected in expatriates who were born in the Emirates. * Mutations reported only in Emiratis. ^‡‡^ Mutations detected in compound heterozygote state.

## Data Availability

This is a research article, and all data generated or analyzed during this study are included in this published article (and its Supplementary Information Files). All inquiries should be directed to the corresponding author’s email: emailnaldewik@hamad.qa; nader.al-dewik@kingston.ac.uk.

## Supplementary Materials

The following supporting information can be downloaded at: https://www.mdpi.com/article/10.3390/biomedicines13092210/s1, Table S1: List of 723 variants (653 SNPs and 70 SVs) associated with BTD deficiency; Table S2: List of 83 variants identified in QGP cohort (n = 14,669), including allele frequencies, homozygous and heterozygous variants; Table S3: Allele frequencies and statistical comparison of BTD variants between QGP and international Population (IP); Table S4: Salt bridge analysis of WT and single mutants of BTD, comparing the number of salt bridges formed in each mutant to the WT structure; Figure S1: Structural validation of the BTD-WT model using ERRAT and ProSA tools. Figure S2: Structural quality assessment of single mutants of BTD using ERRAT analysis. Figure S3: Ramachandran plot analysis of single mutant structures of BTD. Figure S4: Overall structure and catalytic triad of BTD. Figure S5: Electrostatic surface potential analysis of WT and single mutants of BTD. Figure S6: Principal component analysis (PCA) of conformational dynamics in WT and single mutants of BTD. Figure S7: Covariance matrix analysis of conformational dynamics in WT and single mutants of BTD.

## Abbreviations

The following abbreviations are used in this manuscript:

BD	Biotinidase deficiency
BTD	Biotinidase
CADD	Combined annotation-dependent depletion
ESBRI	Electrostatic salt bridge
FEL	Free energy landscape
GRAVY	Grand average of hydropathy
LINCS	Linear constraint solver
MD	Molecular dynamics
NBS	Newborn screening
NMD	Nonsense-mediated decay
PCA	Principal component analysis
PDB	Protein Data Bank
PhD-SNP	Predictor of human deleterious SNPs
PME	Particle mesh Ewald
PolyPhen-2	Polymorphism Phenotyping v2
QGP	Qatar Genome Program
Rg	Radius of gyration
RMSD	Root mean square deviation
RMSF	Root mean square fluctuation
SASA	Solvent-accessible surface area
SIFT	Sorting Intolerant from Tolerant
SNAP	Screening for non-acceptable polymorphisms
SNP	Single-nucleotide polymorphism
SPDB	Swiss Protein Data Bank
SV	Structural variant
