# Supplementary material for: Genomic and Structural Investigation of Mutations in Biotinidase (BTD) Gene Deficiency in Greater Middle Eastern Cohort: Insights from Molecular Dynamics Study
Source: Biomedicines. 2025 Sep 9;13(9):2210. doi: 10.3390/biomedicines13092210 (PMC12467526; doi:10.3390/biomedicines13092210)
Supplement: Supplementary file 1 [file biomedicines-13-02210-s001.zip › biomedicines-3684120_BTD_Figures_S1-S7.pdf]

Genomic and Structural Investigation of Mutations in Biotinidase (BTD) Gene Deficiency in Greater Middle Eastern Cohort: Insights from Molecular Dynamics Study

Faisal E. Ibrahim[1,2,3,†], BalaSubramani Gattu Linga[1,2,†], Muthanna Samara[4], Jameela Roshanuddin[1,2,5], Salma Younes[6,7], Gheyath Nasrallah[6,7], Hatem Zayed[6], M. Walid Qoronfleh[8,*], Sawsan G. A. A. Mohammed[9], Dalia El Khoury[10], Dinesh Velayutham[11], Ghassan Abdoh[12], Hilal Al Rifai[13] and Nader Al-Dewik[1,2,3,5,12,13,14,*]

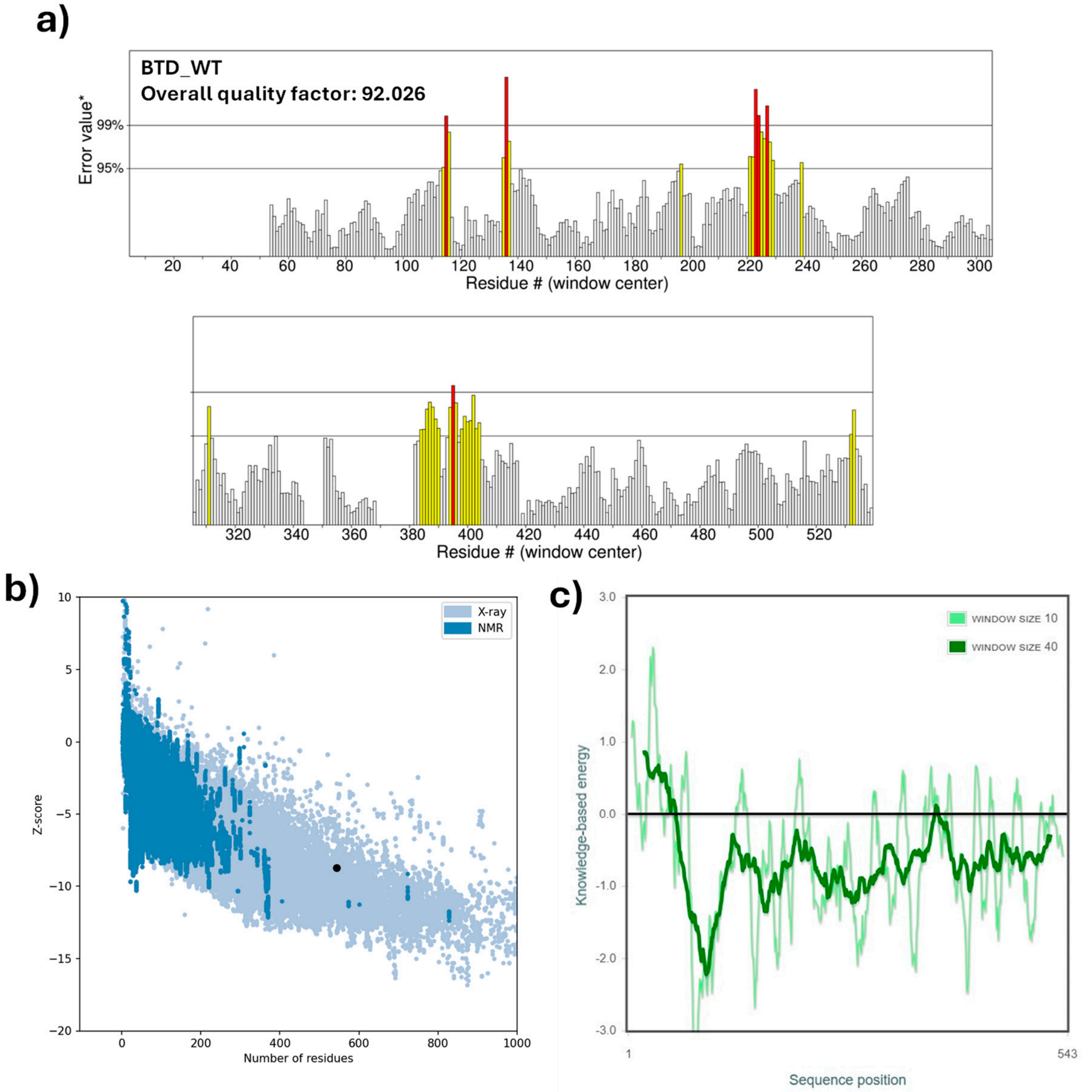

Figure S1. Structural validation of the BTD-WT model using ERRAT and ProSA tools. (a) Structural quality evaluation using the ERRAT program, which identifies potentially problematic regions in the protein model based on non-bonded atomic interactions. Error values are plotted along the sequence, with thresholds at 95% and 99% confidence marked. Regions exceeding these thresholds are highlighted in yellow and red, indicating local structural deviations. (b) ProSA Z-score plot showing the position of the BTD model (black dot) within a database of experimentally determined protein structures. Light and dark blue point clouds represent X-ray and NMR-derived structures, respectively. The BTD model Z-score falls within the range typically observed for high-quality X-ray structures, indicating

favorable overall model quality. (c) Knowledge-based energy profile of the BTD model calculated by ProSA, plotted along the amino acid sequence using sliding window sizes of 10 (light green) and 40 (dark green). Regions with positive energy values correspond to structurally less favorable or potentially erroneous regions of the model.

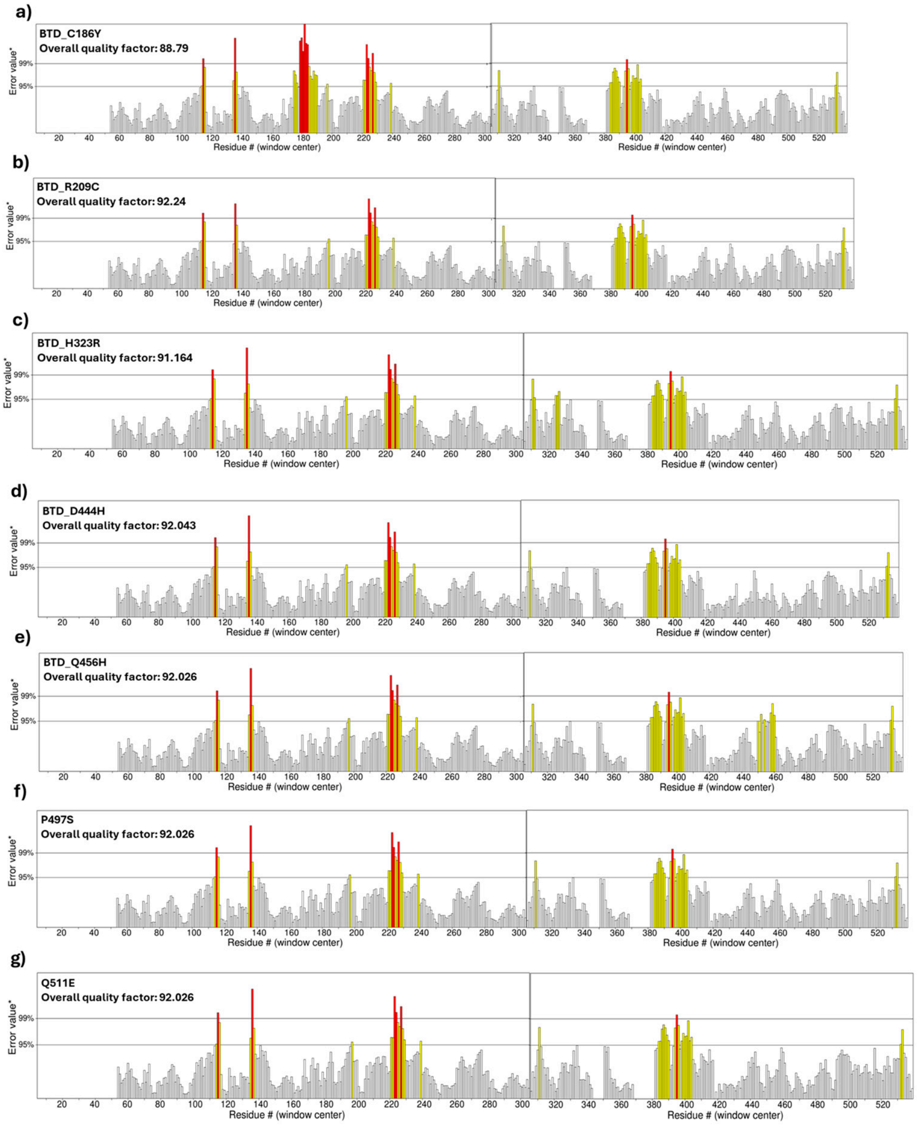

Figure S2. Structural Quality Assessment of Single Mutants of BTD Using ERRAT Analysis. (a-h) The ERRAT program evaluated the overall quality of the single mutants of BTD (a-h) model. On the error axis, two lines (95% and 99%) indicate the confidence with which it is possible to reject regions that exceed that error value. Regions of the structure highlighted in grey and black can be rejected at 95 % and 99 % confidence level, respectively.

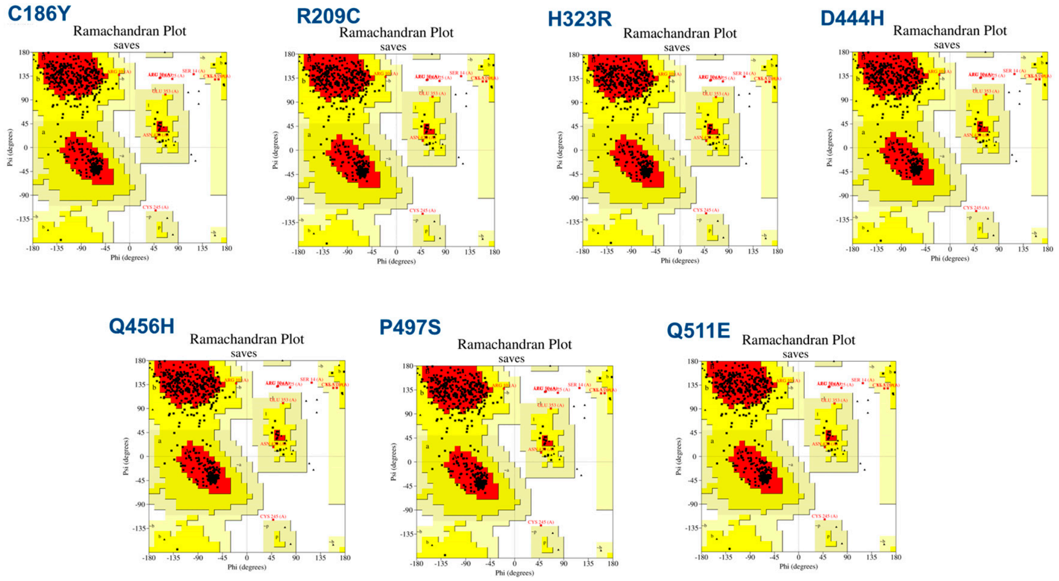

Figure S3. Ramachandran Plot Analysis of Single Mutant structures of BTD. Ramachandran plot analysis of AlphaFold modeled BTD, indicating residues in the favored regions (red), allowed regions (yellow), generously allowed regions (light yellow), and disallowed regions (white) of all single mutant models of BTD.

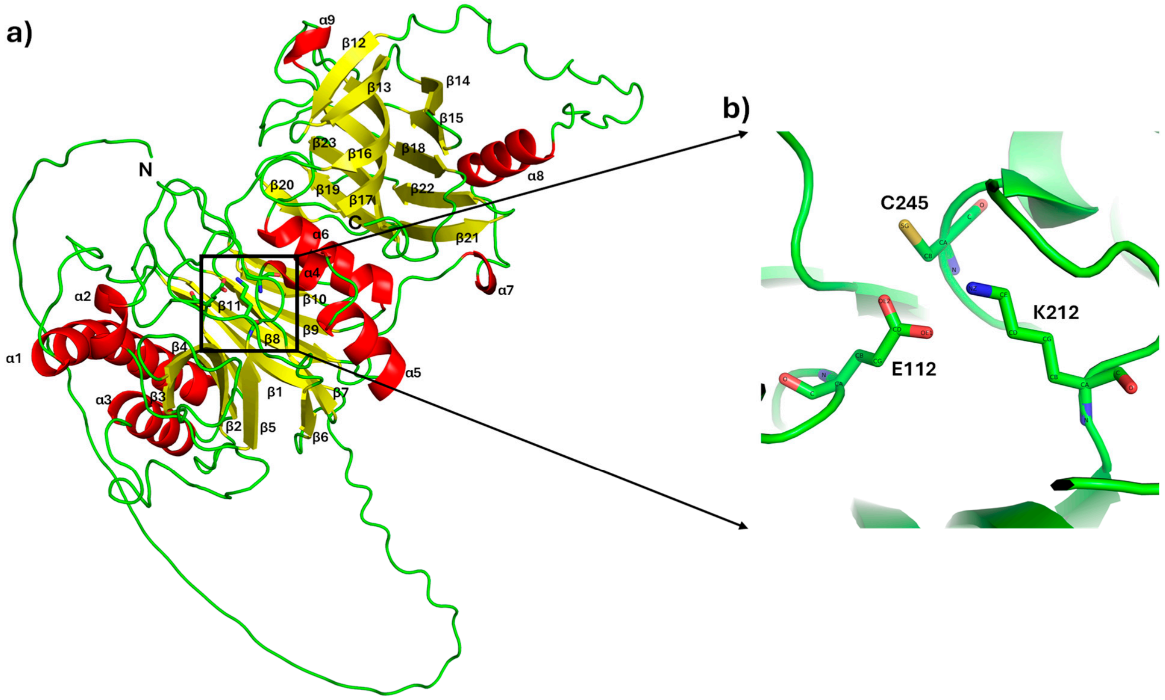

Figure S4. Overall structure and catalytic triad of BTD. a) Structural architecture of apo BTD (representing monomer) with secondary structural elements is indicated. b) The catalytic residues are shown as sticks, indicating their positions - molecular surface presentation of the predicted catalytic pocket of BTD. The active site triad, E112, K212, and C245, is the amidase reaction site of BTD. The nucleophile elbow showing C245 is located in the β-strand-turn-helix structure b) Close-up of the catalytic pocket of the enzyme showing active site triad, E112, K212, and C245, is shown in a ball-and-stick presentation. The carbon, oxygen, nitrogen, and sulfur atoms are colored in green, red, blue, and yellow, respectively.

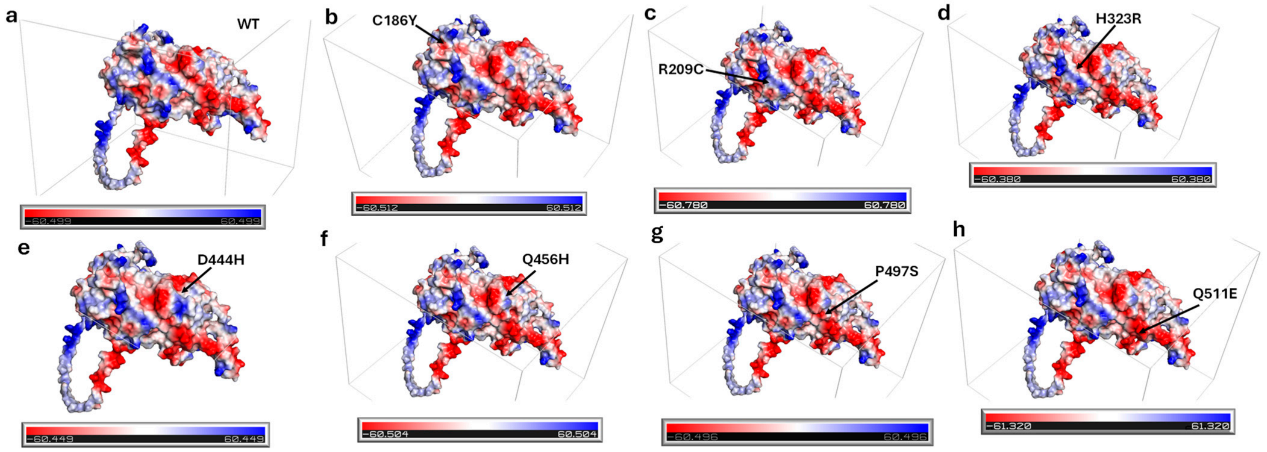

Figure S5. Electrostatic Surface Potential Analysis of WT and Single Mutants of BTD. Electrostatic surface potential of native and single mutants of BTD (a) WT, (b) C186Y, (c) R209C, (d) H323R, (e) D444H, (f) Q456H (g) P497S and (h) Q511E mutants. Red symbolizes a negative charge, blue is positive, and white is neutral. The arrow indicates the biotin binding sites in the BTD.

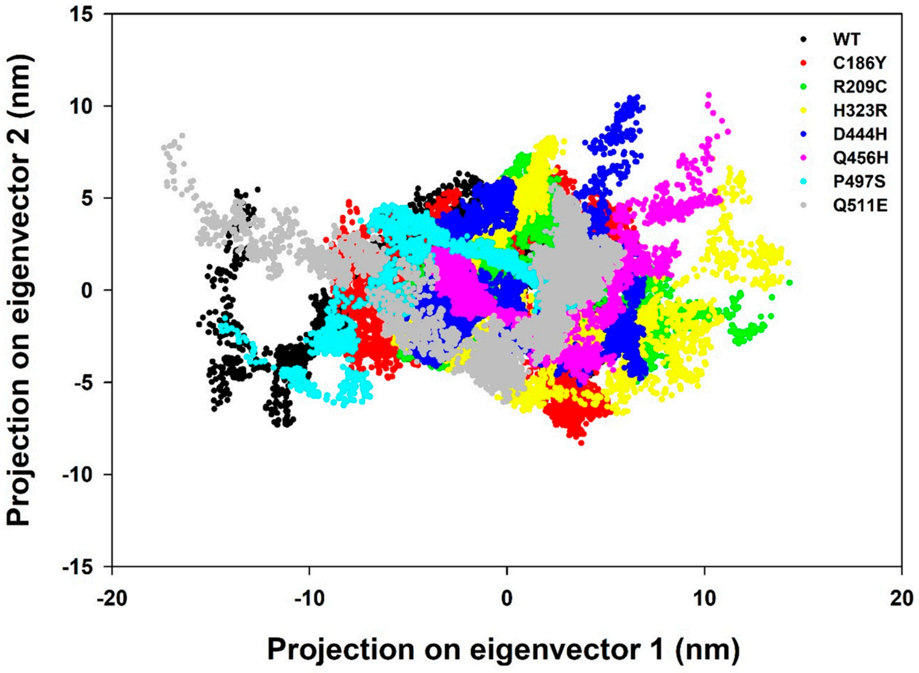

Figure S6. Principal Component Analysis (PCA) of Conformational Dynamics in WT and Single Mutants of BTD. PCA was performed to explore the essential dynamics of the BTD-WT and seven single-point mutants (C186Y, R209C, H323R, D444H, Q456H, P497S, Q511E) at 300 K. The scatter plot displays the projection of molecular motions along the first two principal components (PC1 and PC2), which represent the most dominant collective motions derived from the Cα atomic coordinates. Each colored cluster corresponds to a distinct BTD variant, allowing comparison of the conformational space sampled during simulations. Overlap or separation of clusters reflects the similarity or divergence in dynamic behavior between WT and mutant forms.

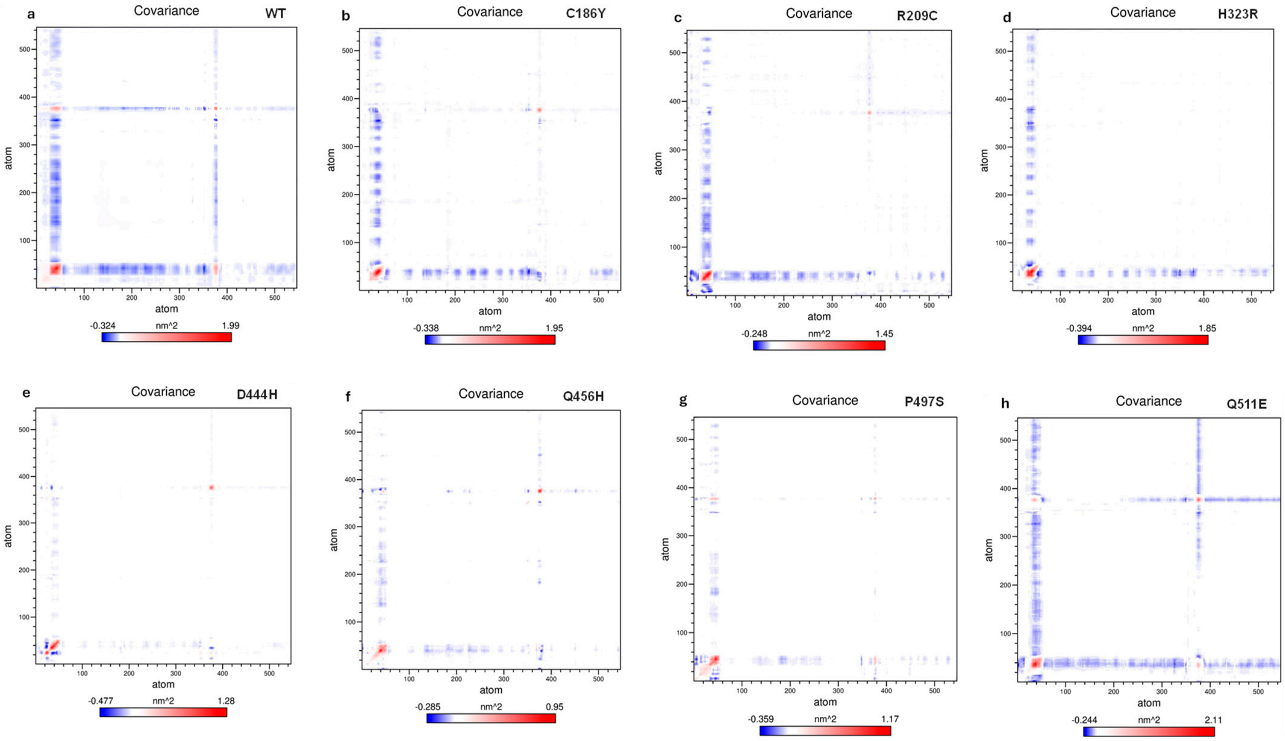

Figure S7. Covariance Matrix Analysis of Conformational Dynamics in WT and Single Mutants of BTD. Computed covariance matrix plots for (a) WT (b) C186Y, (c) R209C, (d) H323R, (e) D444H, (f) Q456H, (g) P497S (h) Q511E mutants of BTD enzyme. Red corresponds to a positive correlation, showing the atom's motion along the same direction, and blue signifies a negative correlation, indicating motion in opposite directions.
