# Supplementary material for: Genomic and Structural Investigation of Mutations in Biotinidase (BTD) Gene Deficiency in Greater Middle Eastern Cohort: Insights from Molecular Dynamics Study
Source: Biomedicines. 2025 Sep 9;13(9):2210. doi: 10.3390/biomedicines13092210 (PMC12467526; doi:10.3390/biomedicines13092210)
Supplement: Supplementary file 1 [file biomedicines-13-02210-s001.zip › biomedicines-3684120_BTD_Table_S4.pdf]

Genomic and Structural Investigation of Mutations in Biotinidase (BTD) Gene Deficiency in Greater Middle Eastern Cohort: Insights from Molecular Dynamics Study

Faisal E. Ibrahim [1,2,3,†], BalaSubramani Gattu Linga [1,2,†], Muthanna Samara [4], Jameela Roshanuddin [1,2,5], Salma Younes [6,7], Gheyath Nasrallah [6,7], Hatem Zayed [6], M. Walid Qoronfleh [8,*], Sawsan G. A. A. Mohammed [9], Dalia El Khoury [10], Dinesh Velayutham [11], Ghassan Abdo [12], Hilal Al Rifai [13] and Nader Al-Dewik [1,2,3,5,12,13,14,*]

Table S4. Salt bridge analysis of WT and Single mutants of BTD, comparing the number of salt bridges formed in each mutant to the WT structure.

**Salt Bridges**		**Distance between residues of native and mutant proteins (Å)**							
Residue 1	Residue 2	WT	C186Y	R209C	H323R	D444H	Q456H	P497S	Q511E
NH1 ARG A 79	OD1 ASP A 130	3.65	3.65	3.65	3.65	3.65	3.65	3.65	3.65
NH2 ARG A 79	OD1 ASP A 130	2.66	2.66	2.66	2.66	2.66	2.66	2.66	2.66
ND1 HIS A 116	OD1 ASP A 113	3.91	3.91	3.91	3.91	3.91	3.91	3.91	3.91
NE2 HIS A 116	OD1 ASP A 113	3.91	3.91	3.91	3.91	3.91	3.91	3.91	3.91
NH1 ARG A 122	OD1 ASP A 222	2.87	2.87	2.87	2.87	2.87	2.87	2.87	2.87
NH1 ARG A 122	OD2 ASP A 222	2.77	2.77	2.77	2.77	2.77	2.77	2.77	2.77
NH2 ARG A 122	OD1 ASP A 189	2.78	2.78	2.78	2.78	2.78	2.78	2.78	2.78
NH2 ARG A 122	OD2 ASP A 189	3.35	3.35	3.35	3.35	3.35	3.35	3.35	3.35
NH2 ARG A 122	OD2 ASP A 222	3.52	3.52	3.52	3.52	3.52	3.52	3.52	3.52
NH1 ARG A 148	OE1 GLU A 145	3.58	3.58	3.58	3.58	3.58	3.58	3.58	3.58
NH1 ARG A 157	OD1 ASP A 91	2.88	2.88	2.88	2.88	2.88	2.88	2.88	2.88
NH2 ARG A 157	OD1 ASP A 91	2.62	2.62	2.62	2.62	2.62	2.62	2.62	2.62
NH1 ARG A 164	OE1 GLU A 94	2.61	2.61	2.61	2.61	2.61	2.61	2.61	2.61
NH2 ARG A 164	OE1 GLU A 94	3.28	3.28	3.28	3.28	3.28	3.28	3.28	3.28
NZ LYS A 176	OD2 ASP A 151	2.74	2.74	2.74	2.74	2.74	2.74	2.74	2.74
NH1 ARG A 191	OD1 ASP A 189	3.67	3.67	3.67	3.67	3.67	3.67	3.67	3.67
NH1 ARG A 191	OD2 ASP A 189	3.2	3.2	3.2	3.2	3.2	3.2	3.2	3.2
NH2 ARG A 191	OD2 ASP A 189	2.77	2.77	2.77	2.77	2.77	2.77	2.77	2.77
NH1 ARG A 209	OE1 GLU A 177	2.64	2.64	-	2.64	2.64	2.64	2.64	2.64
NH2 ARG A 209	OE1 GLU A 177	2.72	2.72	-	2.72	2.72	2.72	2.72	2.72
NH1 ARG A 211	OE1 GLU A 177	3.63	3.63	3.63	3.63	3.63	3.63	3.63	3.63
NH2 ARG A 211	OD1 ASP A 228	2.77	2.77	2.77	2.77	2.77	2.77	2.77	2.77
NH2 ARG A 211	OD2 ASP A 228	3.39	3.39	3.39	3.39	3.39	3.39	3.39	3.39
NZ LYS A 212	OE1 GLU A 112	2.67	2.67	2.67	2.67	2.67	2.67	2.67	2.67
NZ LYS A 212	OE1 GLU A 218	3.7	3.7	3.7	3.7	3.7	3.7	3.7	3.7
NH2 ARG A 239	OD1 ASP A 233	3.43	3.43	3.43	3.43	3.43	3.43	3.43	3.43
NH2 ARG A 239	OD2 ASP A 233	3.24	3.24	3.24	3.24	3.24	3.24	3.24	3.24
NH1 ARG A 256	OD1 ASP A 252	2.86	2.86	2.86	2.86	2.86	2.86	2.86	2.86
NH1 ARG A 256	OD2 ASP A 252	2.74	2.74	2.74	2.74	2.74	2.74	2.74	2.74
NH2 ARG A 256	OD1 ASP A 252	2.98	2.98	2.98	2.98	2.98	2.98	2.98	2.98

NH2 ARG A 256	OD2 ASP A 252	2.74	2.74	2.74	2.74	2.74	2.74	2.74	2.74
NH2 ARG A 259	OD1 ASP A 260	2.9	2.9	2.9	2.9	2.9	2.9	2.9	2.9
NH2 ARG A 259	OD2 ASP A 260	3.54	3.54	3.54	3.54	3.54	3.54	3.54	3.54
ND1 HIS A 302	OE1 GLU A 64	2.74	2.74	2.74	2.74	2.74	2.74	2.74	2.74
NE2 HIS A 358	OE1 GLU A 515	3.53	3.53	3.53	3.53	3.53	3.53	3.53	3.53
ND1 HIS A 447	OD1 ASP A 401	3.37	3.37	3.37	3.37	3.37	3.37	3.37	3.37
ND1 HIS A 447	OD2 ASP A 401	2.75	2.75	2.75	2.75	2.75	2.75	2.75	2.75
NE2 HIS A 456	OD1 ASP A 444	-	-	-	-	-	2.37	-	-
NE2 HIS A 456	OD2 ASP A 444	-	-	-	-	-	3.38	-	-
NH2 ARG A 462	OE1 GLU A 436	2.86	2.86	2.86	2.86	2.86	2.86	2.86	2.86
NH1 ARG A 522	OD1 ASP A 355	3.53	3.53	3.53	3.53	3.53	3.53	3.53	3.53
NH1 ARG A 522	OD2 ASP A 355	2.76	2.76	2.76	2.76	2.76	2.76	2.76	2.76
NH2 ARG A 522	OD1 ASP A 355	2.88	2.88	2.88	2.88	2.88	2.88	2.88	2.88
NH2 ARG A 522	OD2 ASP A 355	2.58	2.58	2.58	2.58	2.58	2.58	2.58	2.58
NZ LYS A 523	OD1 ASP A 510	3.19	3.19	3.19	3.19	3.19	3.19	3.19	3.19
NH1 ARG A 538	OD1 ASP A 543	2.73	2.73	2.73	2.73	2.73	2.73	2.73	2.73
NH1 ARG A 538	OD2 ASP A 543	3.82	3.82	3.82	3.82	3.82	3.82	3.82	3.82
NH2 ARG A 538	OD1 ASP A 543	3.05	3.05	3.05	3.05	3.05	3.05	3.05	3.05
NH2 ARG A 538	OD2 ASP A 543	2.73	2.73	2.73	2.73	2.73	2.73	2.73	2.73
NH1 ARG A 542	OD1 ASP A 444	3.93	3.93	3.93	3.93	-	3.93	3.93	3.93
Total Salt-bridge interaction		48	48	46	48	47	50	48	48
